# Solvation of cationic copper clusters in molecular hydrogen†

**DOI:** 10.1039/d3cp03452f

**Published:** 2023-09-13

**Authors:** O. V. Lushchikova, J. Reichegger, S. Kollotzek, F. Zappa, M. Mahmoodi-Darian, M. Bartolomei, J. Campos-Martínez, T. González-Lezana, F. Pirani, P. Scheier

**Affiliations:** a Institut für Ionenphysik und Angewandte Physik, Universität Innsbruck Technikerstraße 25 Innsbruck 6020 Austria olga.lushchikova@uibk.ac.at; b Department of Physics, Karaj Branch, Islamic Azad University Karaj Iran; c Instituto de Física Fundamental, IFF-CSIC Serrano 123 Madrid 28006 Spain maxbart@iff.csic.es; d Dipartimento di Chimica, Biologia e Biotecnologie, Universita’ di Perugia 06123 Perugia Italy

## Abstract

Multiply charged superfluid helium nanodroplets are utilized to facilitate the growth of cationic copper clusters (Cu_*n*_^+^, where *n* = 1–8) that are subsequently solvated with up to 50 H_2_ molecules. Production of both pristine and protonated cationic Cu clusters are detected mass spectrometrically. A joint effort between experiment and theory allows us to understand the nature of the interactions determining the bonding between pristine and protonated Cu^+^ and Cu_2_^+^ cations and molecular hydrogen. The analysis reveals that in all investigated cationic clusters, the primary solvation shell predominantly exhibits a covalent bonding character, which gradually decreases in strength, while for the subsequent shells an exclusive non-covalent behaviour is found. Interestingly, the calculated evaporation energies associated with the first solvation shell markedly surpass thermal values, positioning them within the desirable range for hydrogen storage applications. This comprehensive study not only provides insights into the solvation of pristine and protonated cationic Cu clusters but also sheds light on their unique bonding properties.

## Abstract 2

We underscore a solid agreement between experimental and computational studies on solvation of pristine/protonated Cu_*n*_^+^ (*n* = 1–2) in H_2_. For instance, with *n* = 1, the initial shell comprises four covalently bound H_2_; subsequent ones are non-covalent.

## Graphical abstract

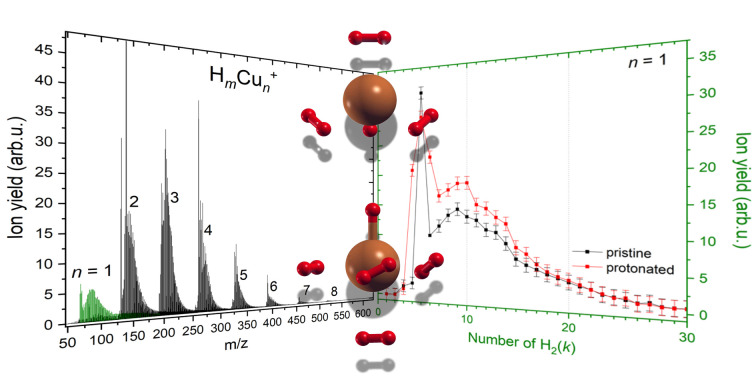

## Introduction

Hydrogen is the most abundant and lightest chemical element in the universe. However, due to its high reactivity, it exists, under ambient conditions on Earth, only as a diatomic molecule or bound to other elements forming compounds such as water.^1^ Nowadays, there is an increasing hydrogen demand, since it is an important chemical species for energy storage, catalysis, metallurgy *etc.* Therefore, great efforts are being made in order to understand the interaction of hydrogen with other elements and molecules.

Copper, on the other side, has proven to be a very robust catalyst for the synthesis of alcohols, hydrocarbons^2–4^ and an interesting candidate for hydrogen storage.^5–9^ In all these processes understanding the interaction between Cu and H_2_ is highly important.

The first attempts were made already in the early 80s and became a computational benchmark for molecule-surface reactions.^10–12^ Ever since, the continuous development of experimental and computational methods led to a good understanding of the reaction between H_2_ and Cu surfaces with different crystalline structures.^13–16^ The dissociation behavior of H_2_ has been summarized in theoretical studies.^17,18^ Moreover, it has been shown that the reactivity decreases from Cu(211) > Cu(111) > Cu(100) > Cu(110), with the last one being rather inert.^19–21^ All types of defects on the surface decrease the H_2_ dissociation barrier. Involved defects were modelled with gas-phase Cu clusters to reduce the complexity of the system and to focus on the reaction between Cu and H_2_.

However, the size of clusters can significantly impact their physical and chemical properties, such as the height of the dissociation barrier of the molecules attached to this cluster. Research on Ag clusters demonstrated that the size of deposited clusters is even more important for its catalytic activity than the underlying support material.^22^ H_2_ binds also more strongly to the clusters than to the surfaces, and it was recently shown that H_2_ takes more charge from Cu_13_ than from a Cu(111) surface.^13^

The reaction of neutral Cu clusters with H_2_ has been studied mainly theoretically.^23–32^ It was found that the chemisorption of hydrogen on clusters more likely happens on acute metal sites, in contrast to surfaces where it usually binds to hollow sites.^27^ The adsorption of the first H_2_ molecule, in general, leads to the reduction of the Cu–Cu distance and elongation of the H–H bond lengths.^26^ Moreover, the chemisorption energy of the first hydrogen atom exhibits strong cluster size-dependent even–odd oscillations, favoring the clusters with an even number of Cu atoms.^33^ Experimentally, the reaction probability of D_2_ with Cu clusters was measured at single-collision-like conditions. In that study, it was concluded that the reactivity of Cu is so low, that it is below the detection limit.^34^

In contrast, Cu cations have attracted more attention among experimentalists, supported by theoretical investigations.^35–39^ Among other transition metal ions, the reaction product of Cu^+^ with H_2_ has been extensively studied with IR spectroscopy.^40^ Dissociation energies for the detachment of up to four H_2_ molecules, forming the first solvation shell, from Cu^+^ (0.67, 0.72, 0.38 and 0.22 eV) and from the electronically excited Cu*^+^ (0.18, 0.11 and 0.06 eV, first three ligands) have been obtained using equilibrium methods.^36^ Similar energies were determined for Cu_2_^+^ (0.54, 0.44, 0.21, 0.16, 0.09 and ∼0.07 eV).^37^ Recently, bigger cationic clusters consisting of *n* = 4 up to 7 Cu atoms have been also investigated using IR-spectroscopy, showing that clusters with *n* = 5 have a tendency for dissociative adsorption of H_2_, while other cluster sizes exhibit mainly molecular adsorption.^41^ It has been found that not more than six H_2_ ligands could be strongly attached to the cation.^37,41^

Previously, multiple solvation layers of hydrogen molecules surrounding H^−^ ^42^ and H_3_^+^ ^43^ were studied by electron impact of neutral superfluid He nanodroplets (HNDs) heavily doped with H_2_, as well as the solvation of cationic impurities such as fullerenes^44^ and Cs^+^ ^45,46^ by H_2_. In the present investigation, by utilizing multiply charged superfluid He nanodroplets (mc-HNDs) we were able to grow Cu_*n*_^+^ (*n* = 1–8) solvated with up to 50 H_2_ molecules, which became possible due to the ultracold environment of the host droplet. A similar method was also recently used to study solvation of Na^+^.^47^ One of the main novelties of this work is that both pristine and protonated cationic Cu clusters are produced. Here we present a joint effort between experiment and theory in order to understand the nature of the interaction determining the bonding in the complexes containing Cu^+^ and Cu_2_^+^ cations and molecular hydrogen. These ionic complexes formed in mc-HNDs are studied by mass spectrometry and their structure and energy are evaluated by means of high-level *ab initio* electronic structure, classical optimization evolutionary algorithm (EA) and diffusion Monte Carlo (DMC) calculations. Further, we also present the experimental results on the solvation of Cu_*n*_^+^ (*n* = 3–8) with H_2_ and collision-induced dissociation (CID) studies of selected complexes.

Obtained results are of relevance from both an applied (hydrogen storage and release) and a fundamental point of view. Particular attention is addressed to the gradual transition between a prevalent covalent bonding, which governs the energy and structure of the first solvation shell, found for both Cu monomer and dimer cations, to that of an exclusive non-covalent behavior which determines the intermolecular interaction within the following shells.

## Methods

### Experimental

The data described in the following were obtained by doping of positively charged HNDs, formed *via* supersonic expansion of pre-cooled ^4^He gas (99.9998%, Messer) at a temperature of 9 K and a stagnation pressure of 25 bar through a 5 μm pinhole nozzle (Plano GmbH, A0200P). Right after passing a skimmer into the quadrupole chamber (QUAD) the HNDs are ionized with electron impact (current 350 μA and energy 60 eV). The resulting mc-HNDs have a size of approximately 10^6^ He atoms and an internal temperature of ∼0.4 K carrying about 10 charges.^48^ The Cu_*n*_^+^ clusters are formed by evaporation of monoisotopic ^63^Cu pellets (99,9% isotopic purity) in the sublimation source (125 W, ∼1020 K measured on the outside surface of the oven) and sequential pick up of Cu atoms into mc-HNDs, where they agglomerate around the charge centers.^49^ A more detailed description of the instrumentation and cluster formation can be found elsewhere.^50,51^

**Fig. 1:**
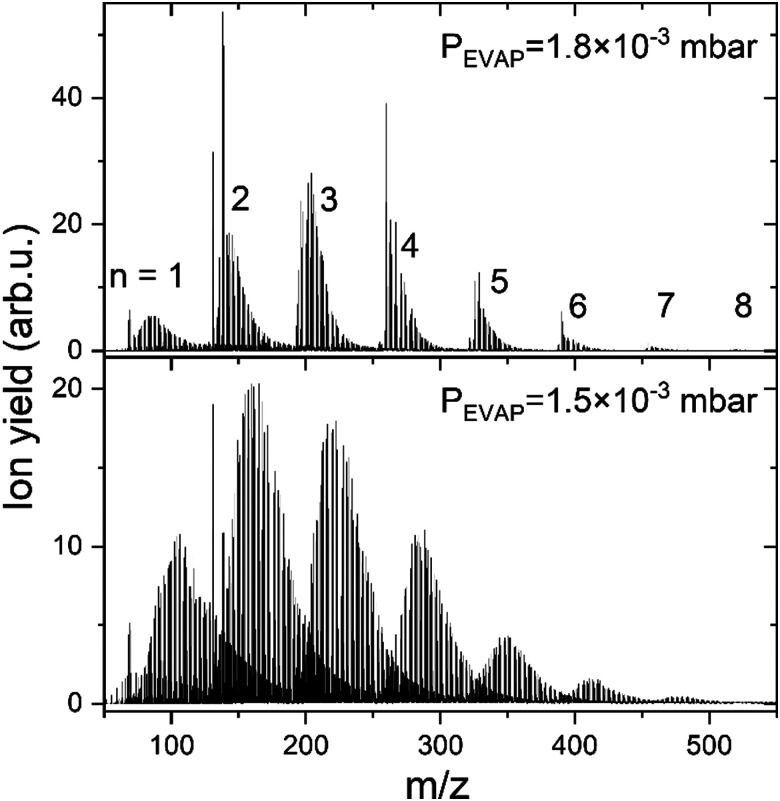
Mass spectra showing H_*m*_Cu_*n*_^+^ ions extracted from multiply charged HNDs doped with ^63^Cu *via* multiply collisions with H_2_ gas at a pressure of 1.8 × 10^−3^ (top) and 1.5 × 10^−3^ (bottom) mbar. The number *n* of Cu atoms in the ions is indicated by the numbers on top of the pronounced peak series.

The Cu_*n*_^+^ (*n* = 1–8) doped HNDs are guided by an RF-hexapole (EVAP) filled with H_2_ gas, as it was also done previously for alkali cations.^47^ In the EVAP chamber He from the HNDs evaporates due to the high number of collisions with room temperature H_2_ (99.999%, Messer). Additionally, the Cu clusters react with H_2_ and exothermic processes also lead to He evaporation from the HND. However, too many collisions also lead to the evaporation of H_2_ units from the H_*m*_Cu_*n*_^+^ complexes, as a consequence less H_2_ remains bound to the copper cluster ions. Therefore, the H_2_ pressure in the EVAP chamber is critical for the amount of H_2_ remaining attached to the copper cluster ions. Here, we will discuss only experiments performed at a pressure of 1.8 × 10^−3^ mbar, since this pressure regime allows to resolve the behavior of the complexes with less than 15 H_2_ molecules attached to Cu_*n*_^+^. This range is of special interest in this study, since it allows the comparison with computational results. The resulting hydrogen-solvated copper cluster ions were analyzed and detected with a time-of-flight mass spectrometer (TOF-MS) to obtain the ion yield of H_*m*_Cu_*n*_^+^/(H_*2*_)_*k*_Cu_*n*_^+^ complexes as a function of the number of H_2_ molecules, *k*, (or H atoms, *m*) bound to a specific copper cluster cation Cu_*n*_^+^. However, also a measurement at 1.5 × 10^−3^ mbar is performed to verify that intensity anomalies are independent of hydrogen pressure in the evaporation cell. The resulting mass spectra are illustrated in Fig. 1. Comparing those spectra, one could notice that as the hydrogen pressure in the evaporation cell decreases, the number of attached H_2_ increases. To facilitate the data analysis the mass spectra were analyzed with the in-house software IsotopFit.^52^

Additionally, protonated Cu_*n*_^+^ are studied during the same measurement. HCu_*n*_^+^ core ions are produced *via* proton transfer from H_3_^+^. The backflow of H_2_ from the EVAP chamber to the QUAD chamber results in the formation of H_3_^+^ ions in the mc-HND prior to copper pickup, which transfer a proton to the first arriving copper atom.^50^ Therefore, the solvation of Cu_*n*_^+^ and HCu_*n*_^+^ in H_2_ can be studied simultaneously. The ion intensity distributions of H_*m*_Cu_*n*_^+^ ions are extracted from the mass spectra in Fig. 1 and are plotted as a function of the attached H atoms *m* for clusters containing up to *n* = 8 copper atoms in Fig. S1 in ESI,† for both studied pressures. Note that all figures and tables started with S, can be found in ESI.†

To verify that both Cu_*n*_^+^ and HCu_*n*_^+^ are formed during protonation, additional mass spectra were recorded. In this case, the H_2_ gas is introduced to the QUAD chamber at a pressure of 9.6 × 10^−7^ mbar, which corresponds to the pressure in this chamber during the solvation studies described above. However, in contrast to the previous measurements, the shrinking of the He droplet is done by collisions with room temperature He rather than H_2_. Fig. S2 (ESI†) clearly shows the presence of H_*m*_Cu_*n*_^+^ ions with both even and odd numbers of H atoms. Nevertheless, this method does not allow the attachment of more than ten H atoms to the cluster even at the highest acceptable hydrogen pressure of 2 × 10^−6^ mbar. Higher solvation of ions with H_2_ can be done only in the EVAP chamber, where due to the differential pumping a pressure of at least three orders of magnitude higher than that allowed in the QUAD chamber can be reached.

The results of cluster solvation by H_2_ were confirmed by measurements under slightly different experimental conditions with H_2_ and D_2_ (99.999%, Linde) for Cu_*n*_^+^ (*n* = 1–5). The overall trend in the distributions occurred to be very similar. A comparison between two hydrogen and one deuterium measurements is illustrated in Fig. S3 and experimental conditions are listed in Table S1 (ESI†). The distributions illustrated in the main text of this paper for *n* = 1 and 2, were obtained with experimental setting referred as “H_2_ small clusters” in the Table S1 (ESI†).

For selected complexes, CID measurements were performed to reveal the fragmentation paths. After the formation of H_*m*_Cu_*n*_^+^ the desired complex was selected with an additional RF-quadrupole mass filter and then guided into a collision cell, which is an RF-hexapole ion guide inside a differentially pumped cylinder containing argon at a pressure of 1 × 10^−5^ mbar. For this experiment D was used rather than H to increase the accuracy of the experiment, due to the limited resolution of the mass filter for selecting only one specific precursor ion. The collision energy was varied from 1 to 60 eV, in the lab energy frame, *e.g.* the kinetic energy applied to the original ionic complex prior to collisions. Therefore, further in the text all reported energy values refer to the same energy. Please note that the complex undergoes multiple collisions during CID. As a result, the findings are purely qualitative in nature, precluding any definitive conclusions about binding energies. The resulting fragments are also detected with the TOF-MS as described previously.

### Computational

The most stable structures and related energies of the smaller (H_2_)_*k*_Cu_*n*_^+^ and (H_2_)_*k*_HCu_*n*_^+^ (*n* = 1–2) clusters have been obtained at the CCSD level of theory together with the aug-cc-pVDZ^53^ basis set by means of a geometry optimization (and related frequencies calculation) procedure as implemented in the Gaussian 09 computational package.^54^ For each cluster several initial geometries have been investigated in order to identify those structures leading to minima for the interaction energy corresponding to the adsorption of *k* H_2_ molecules. Those interaction energies have been obtained at the CCSD(T) level and in the complete basis set (CBS) limit by exploiting the two-point correlation energy extrapolation of Halkier *et al*.^55,56^ The latter consists in properly combining counterpoise corrected^57^ CCSD(T) and Hartree–Fock energies obtained with the aug-cc-pVDZ and aug-cc-pVTZ basis sets.

**Fig. 2:**
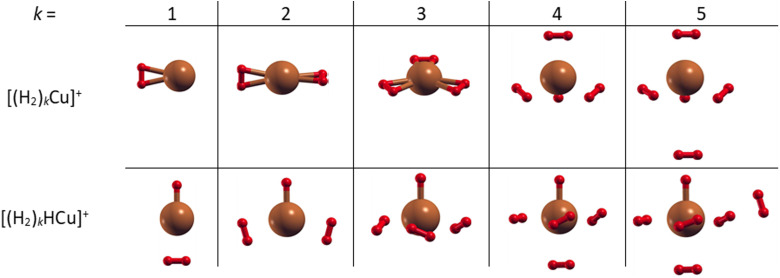
Global minima structures as obtained by means of geometry optimizations at the CCSD/aug-cc-pVDZ level of theory for the (H_2_)_*k*_Cu^+^ and (H_2_)_*k*_HCu^+^ (*k* = 1–5) clusters. In both cases, up to four H_2_ molecules tend to gather around the Cu and HCu cores to form a first solvation shell. Notice that the Cu–H bonds depicted as solids are by convention those with an internuclear distance of less than 1.815 Å.

The obtained structures, corresponding to the putative global minima of the (H_2_)_*k*_Cu^+^ and (H_2_)_*k*_HCu^+^ (*k* = 1–5) clusters, are reported in Fig. 2: it can be appreciated that in both cases we have up to four molecules which arrange themselves around and closer to the Cu^+^ (or HCu^+^) species. In particular, as observed in Fig. S4 (ESI†), the separation of the H_2_ centers of mass from the metal atom varies from about 1.73 to 1.85 Å for (H_2_)_*k*_Cu^+^ (*k* = 1–4) and from 1.80 to 2.10 Å for (H_2_)_*k*_HCu^+^ (*k* = 1–4), while, if an additional H_2_ molecule is added, it tends to locate farther from the Cu atom at about 3.3 Å, as noticed for the cases with *k* = 5. Moreover, it has been checked that the geometry of the four inner H_2_ molecules is very little affected by the presence of a fifth one. Therefore, it can be suggested that a first solvating shell, composed of four H_2_ molecules, should be found for both clusters’ classes and a confirmation of this hypothesis will be proposed in the next section from the analysis of the related interaction and evaporation energies.

**Fig. 3:**
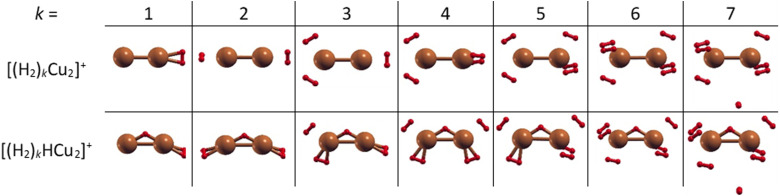
Global minima structures as obtained by means of geometry optimizations at the CCSD/aug-cc-pVDZ level of theory for the (H_2_)_*k*_Cu_2_^+^ and (H_2_)_*k*_HCu_2_^+^ (*k* = 1–7) clusters. In both cases up to six H_2_ molecules tend to gather around the Cu_2_ and HCu_2_ cores. Notice that the Cu–H bonds depicted as solids are by convention those with an internuclear distance of less than 1.815 Å.

The structures of the putative global minima of the (H_2_)_*k*_Cu_2_^+^ and (H_2_)_*k*_HCu_2_^+^ (*k* = 1–7) clusters are shown in Fig. 3 where it can be appreciated that up to six H_2_ molecules tend to evenly gather around both Cu atoms and close to them. In particular, as observed in Fig. S5 (ESI†), the Cu–H_2_ separation varies in the 1.77–1.86 Å and 1.71–1.90 Å ranges for the (H_2_)_*k*_Cu_2_^+^ and (H_2_)_*k*_HCu_2_^+^ (*k* = 1–6) aggregates, respectively. If an additional H_2_ molecule is added, it tends to place itself farther from the related Cu atom at about 3.6 Å, as noticed for the cases with *k* = 7. Therefore, this suggests that for the clusters involving (Cu_2_)^+^ and (HCu_2_)^+^ species the first solvation shell should include six H_2_ molecules as it will discussed in the next section.

As for larger solvation shells, for the sake of simplicity, we have decided to focus only on the (H_2_)_*k*_Cu^+^ aggregates, characterized by a closed shell character of the impurity, *i.e.* Cu^+^, and being therefore more easily treatable by means of high level electronic structure calculations.

For such aggregates we have developed an analytical potential energy surface (PES) capable to correctly describe and represent the involved non-covalent interaction.

In particular, since as seen above for the most stable configuration of the (H_2_)_5_Cu^+^ aggregate the fifth H_2_ molecule is located farther from the Cu atom of about 1.5 Å (with respect to the inner H_2_ shell) and more loosely bound (see next section), we believe that it is a good approximation to obtain a PES which takes into account only for the interaction only between an external H_2_ molecule and the inner (H_2_)_4_Cu^+^ core, which has been considered as a rigid body. Such a PES has been built in a similar way to that recently reported for the (He_*k*_-SF_5_)^+^ and (He_*k*_-SF_6_)^+^ clusters.^58^

The structure of the (H_2_)_4_Cu^+^ cation has been optimized as explained above and it consists in a tetrahedral-like geometry with the Cu atom in the center and four H_2_ molecules constituting the inner shell located around the metal and at about 1.83 Å from it.

Moreover, the related partial atomic charges obtained at the CCSD/aug-cc-pVTZ level of theory through the CM5 approach^59^ have revealed that most of the cation positive charge is borne by the Cu atom while small (non negligible) charges can also be associated to the remaining H atoms.

As a following step, accurate *ab initio* estimations of the H_2_-(H_2_)_4_Cu^+^ interaction potential were performed at the CCSD(T) level by considering both monomers as rigid bodies. In particular, the (H_2_)_4_Cu^+^ minimum geometry is that detailed above while the H_2_ internal distance |*r*| is fixed at its averaged value for the ground vibrational state *r*_0_ = 0.766638 Å.

**Fig. 4:**
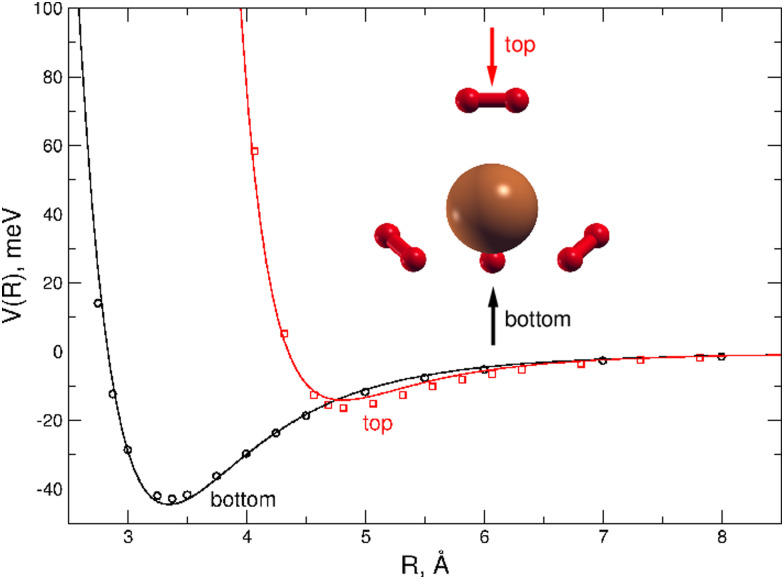
Interaction potential cuts (in meV) for two different approaches (shown with the red and black arrows) of the H_2_ molecule along the main symmetry axis of the (H_2_)_4_Cu^+^ cation. Open circles correspond to the reference CCSD(T)/CBS estimations while the solid lines to the analytical PES. Red and black profiles correspond to the “top” and “bottom” approaches, respectively, and each profile is the average of three potential energy curves obtained for the two principal perpendicular and one parallel orientations of the H_2_ molecules with respect to cation main axis. The intermolecular distance *R* (in Å) is here that from the Cu atom and the center of mass of H_2_.

A dense grid along the intermolecular coordinate *R* defining the distance between the external H_2_ unit and the Cu atom of the (H_2_)_4_Cu^+^ core, was probed for two selected approach configurations of the diatomic molecule along the main symmetry axis of the cation, as shown in Fig. 4. For both “bottom” and “top” approaches three different orientations (the main two perpendicular and one parallel) of the H_2_ molecules with respect to the cation axis has been considered: the corresponding interaction energies have been then combined in order to obtain an averaged interaction which can be considered as a good prediction in the limit of considering the H_2_ monomer as a pseudoatom. This approach is suggested by the sufficient large separation distance of H_2_ from the center of (H_2_)_4_Cu^+^ cation, since under such conditions its orientation anisotropy tends to vanish. We believe that this approximation is reasonable to correctly describe the clusters under study when further H_2_ molecules are added to the more strongly bound first solvation shell. In order to estimate the computed interaction energies in the CBS limit we have applied the same procedure mentioned above which combines counterpoise corrected^57^ CCSD(T)/aug-cc-pVTZ energies with those at the Hartree–Fock and MP2 levels and obtained with two contiguous aug-cc-pVXZ (X = T,Q) basis sets.

All interaction energies reported in Fig. 4 are defined as the energy difference between the cluster and infinitely separated monomers having the same geometry than in the complex. They have been obtained by using the Molpro2012.1 computational package.^60^

The *ab initio* interaction energies have then served as reference values for the optimization of the analytical representations of the PES. The total interaction between the (H_2_)_4_Cu^+^ ion and an external H_2_ (treated as a pseudoatom) can be formulated as a combination of two components

1 *V*_total_ = *V*_vdW_ + *V*_ind_,

which represent the van der Waals (size repulsion plus dispersion attraction) and induction contributions, respectively.

The van der Waals *V*_vdW_ term is expressed as a sum of atom (on (H_2_)_4_Cu^+^)-external (pseudo) atom pair-wise contributions2
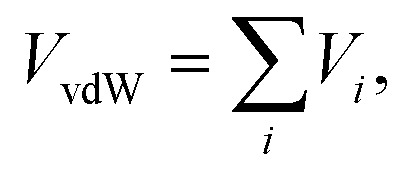
where the sum runs over all possible atoms on the (H_2_)_4_Cu^+^ monomer.

The formulation adopted for each term *V*_*i*_ term in eqn (2) is of the Improved Lennard Jones (ILJ) type:^61^3
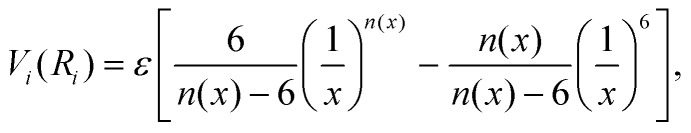
where *x* is the reduced distance of the two bodies defined as4
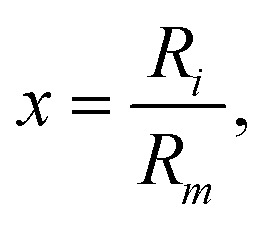
with *R*_*i*_ being the distance between the *i*^th^ atom on (H_2_)_4_Cu^+^ and the outer H_2_ molecule (described as a pseudoatom) while *ε* and *R*_*m*_ are, respectively, the well depth and its position of the interaction potential at the equilibrium value of *R*_*i*_.

The *n*(*x*) exponential parameter allows the ILJ functional form to have an additional flexibility with respect to the usual Lennard-Jones(12,6) (LJ) ones thanks to its dependence on *R*_*i*_:^61^

5 *n*(*x*) = *β* + 4.0*x*^2^,

in which *β* is a parameter depending on the nature and the hardness of the interacting particles leading to a more realistic representation of both repulsion (first term in square brackets of eqn (3)) and attraction (second term in square brackets of eqn (3)).

As for the *V*_ind_ term of eqn (1), it has been introduced to describe the attractive charge-induced dipole contribution determined by the integer positive charge on the (H_2_)_4_Cu^+^ cation. In order to simplify the expression of the *V*_ind_ term, and considering that as anticipated above most of the effective positive charge is borne by the Cu atom, we have formulated it as depending by the square of the field generated only by such a charge:6
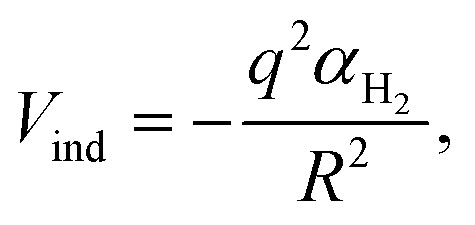
where *α*_H_2__ is the static dipole polarizability of the external H_2_ molecule (whose isotropic component amounts to 0.79 Å^3^) while *q* is the effective charge associated to the Cu atom.

The parameters involved in eqn (3)–(6) were fine-tuned through the comparison between total interaction potential *V*_total_ and the reference *ab initio* estimations. The resulting optimized potential parameters are gathered in Table 1 as well as in Table S2 (ESI†) where the optimized geometry of (H_2_)_4_Cu^+^ is reported together with the related charge distribution.

**Table 1:** Optimized parameters for the van der Waals contribution (*V*_vdW_) to the H_2_-[(H_2_)_4_Cu]^+^ PES (see eqn (3)–(5)). Parameters for the induction contribution (see eqn (6)) are indicated in the ESI. *R*_*m*_ are given in Å and *ε*, in meV; *β* is dimensionless

Pair	H_2_[(H_2_)_4_Cu]^+^		
	*R*_*m*_	*ε*	*β*
H_2_–Cu	3.822	4.98	6.1
H_2_–H	3.250	2.11	6.1

A comparison between the analytical PES and the CCSD(T) calculations is presented in Fig. 4 where it can be appreciated that the present analytical representation is capable to provide a very good agreement with the reference *ab initio* interaction energies for both the “top” and “bottom” approaches, the latter being the most attractive profile with a well depth of about 44 meV at an intermolecular distance around 3.35 Å. Due to the tetrahedral-like symmetry of the inner cation three additional minima, having very similar interaction features as that found for the “bottom” approach, are indeed also expected, as we will see in the following.

In order to describe the energy and structure of the (H_2_)_*k*_Cu^+^ (with *k* > 5) clusters, the H_2_–H_2_ interaction must be also taken into account and the latter is expressed as a sum of non-covalent plus electrostatic contributions, following the procedure indicated in the work by Kranabetter *et al.*^46^

As for three-body contributions to the total interaction energy, we have decided to neglect them for the (H_2_)_*k*_Cu^+^ (with *k* > 4) since the main term, that is the charge-induced dipole induced dipole contribution, provides very little corrections due to the larger distance of the outer H_2_ molecule from the central metal atom (bearing most of the cationic charge) with respect to those composing the first solvation shell.

## Results and discussion

### H_*m*_Cu^+^ clusters

**Fig. 5:**
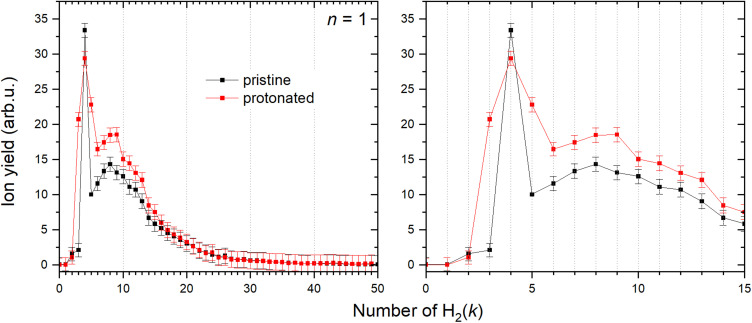
Left: Ion abundances of Cu^+^ (black) and HCu^+^ (red) solvated in the H_2_ with corresponding error bars extracted from the measured mass spectrum. Right: Zoom in on the complexes with first 15 H_2_ molecules attached.

It has been shown^36^ that a copper cation forms with the first H_2_ molecule a bond with an evident partial chemical character. The interaction with the second H_2_ molecule is even stronger, and then the binding energy gradually decreases for the attachment of additional molecules. However, the determination of the corresponding binding energies has been previously obtained only for the first six H_2_ attached to Cu^+^.^36^ Utilizing mc-HNDs we have managed to attach more than 50 H_2_ molecules to Cu^+^. In line with the previous findings, it becomes evident from Fig. 5 that the first stable configuration with abnormally high intensity involves the binding of two H_2_ molecules, a state we term “magic” henceforth. The next magic ion is formed by the attachment of four H_2_ molecules to Cu^+^, providing the first solvation shell. The second solvation shell is formed by *k* = 8. When Cu^+^ is protonated forming the HCu^+^ core, also four H_2_ molecules are required to form the first solvation shell as shown in Fig. 5, followed by shell closures at *k* = 9 and 13. The allocation of magic numbers adheres to the second difference method, expounded upon in our earlier publication.^62^ Additionally, the magic-numbered ions generated by exploring other cluster sizes, *n* = 1–8, and *k* values below 15, are determined in a similar fashion and presented in Table 2.

**Table 2:** Putative magic numbers are listed for complexes formed with pristine and protonated clusters containing *n* = 1–8 copper atoms. Please note that *k* represents number of H_2_ molecules bound to the cluster

Cu_*n*_^+^, *n*	Pristine, *k*	Protonated, *k*
1	2, 4, 8	4, 9, 13
2	6	2, 6, 8
3	4, 7, 9	4, 6, 11
4	4, 6, 11	5, 7
5	4, 7	5, 7
6	6	2, 6, 10, 12
7	4, 7	7
8	7, 12, 14	2, 8, 13

It is known that some cluster sizes are able to dissociate H_2_,^41^ however is very unlikely at the low temperature of the host helium droplet. The appearance in the mass spectrum of lines related to odd *m* can be thus rationalized by the formation of the HCu^+^ core, which is further solvated by H_2_. This is in striking contrast to related experimental findings^45,47^ for similar clusters formed in HND such as (H_2_)_*k*_Na^+^ and (H_2_)_*k*_Cs^+^ for which the HNa^+^ and HCs^+^ species have been hardly detected due to their very low abundance, probably related to the non-chemical character of the alkali metal ion-hydrogen atom bond. Note that alkali ions are closed shell species, with empty orbital much higher in energy compared to those of a Cu ion. Therefore, their interactions with other partners are dominated by nearly pure electrostatic and polarization effects.

The experimental detection of both Cu^+^ and HCu^+^ ionic cores can be explained by the existence of two specific pathways. The first pathway would follow a charge transfer from the HND to Cu with a subsequent addition of molecular hydrogen. A second pathway would involve H_3_^+^ as the first charge carrier and this species can further encounter Cu with the following outcomes,(1) H_3_^+^ + Cu → H_2_^+^ + HCu Δ(*E*) ∼ 2.3 eV(2) H_3_^+^ + Cu → H_2_ + HCu^+^ Δ(*E*) ∼ −3.3 eVIt can be seen that the theoretical electronic energy difference Δ(*E*), obtained from reactants and products optimizations at the CCSD/aug-cc-pVTZ level of theory, clearly favors the second reaction channel promoted by H_3_^+^. Indeed, the optimized products’ structure is shown in Fig. 2 and it is found that HCu^+^ is a chemical species, with a Cu–H bond length and a dissociation energy of about 1.47 Å and 3.0 eV, respectively.

The existence of a chemical bond in HCu^+^ is indeed a consequence of the electron configuration of the Cu^+^ cation whose ground state of a closed shell character reads as 3d.^10^ However, for the first excited singlet state, with the 4s^1^3d^9^ electron configuration and an open shell character, the formation of a covalent bond with a free H atom is energetically favorable. Moreover, such electron configurations with occupied 3d and unoccupied 4s and 4p orbitals are probably also responsible for the optimized geometries found for the (H_2_)_*k*_Cu_*n*_^+^ and (H_2_)_*k*_HCu_*n*_^+^ (*n* = 1–2) clusters and which are discussed in the following.

**Fig. 6:**
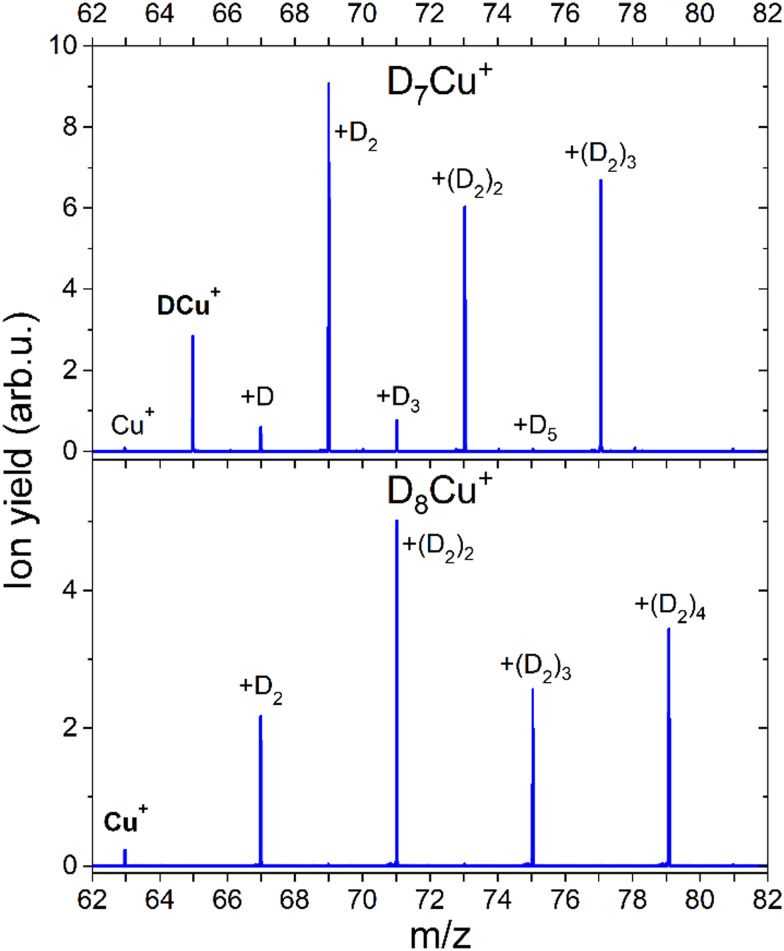
Mass spectra obtained with CID of D_7_Cu^+^ and D_8_Cu^+^ at 10 eV and Ar pressure of 1 × 10^−5^ mbar. The core ion is marked with the bold text. All further labels indicate how many D atoms are attached to the core ion.

The formation of a chemical HCu^+^ species has been further proven by CID measurements illustrated in Fig. 6 where results on deuterated complexes are reported to ensure a better selection of the parent peak: the D_*m*_Cu^+^ clusters with *m* = 7 and *m* = 8 were separately selected and dissociated at the energy of 10 eV in the presence of Ar gas. For the *m* = 7 complex, we mainly see the detachment of D_2_ units until DCu^+^ is formed, while for the *m* = 8 aggregate the final product is Cu^+^. Interestingly, CID of the complex with *m* = 7 also shows very weak peaks at *m* = 0, 2, 4 and 6 suggesting that there is a small probability that single D atoms can detach from the parent peak. Additional measurements were done for *m* = 7, 12, 13 and 14 at 0, 1 and 10 eV and the corresponding mass spectra can be found in ESI,† Fig. S6. In all cases we see similar patterns, complexes with even *m* complexes showing exclusively the loss of D_2_, while odd *m* precursor species exhibiting a preferential D_2_ loss but also low-intensity peaks for fragment ions corresponding to the detachment of single D atoms. From these additional measurements, it is also clear, that D_2_ molecules are weakly bound to the cluster, since they readily dissociate already with 1 eV of collision energy. The only difference between the mass spectra measured at collision energies of 1 and 10 eV is that for the precursors with *m* = 7, 13 the intensity of the fragments with even *m* increases with the collision energy. The appearance of these peaks can be attributed to the neighboring masses “leaking” through the quadrupole filter. As a result, fragments unrelated to the parent ion can emerge in the mass spectrum. It could also originate from the presence of the H_2_ impurity, which mass equals to one D atom. This impurity might stem from the residual gas, as the pumping speed for hydrogen is very low. Additionally, there is a presence of 100 ppm H_2_ in the D_2_ cylinder, as indicated by the supplier. Another possibility is that a small fraction of (D_2_)_*k*_DCu_*n*_^+^ complexes can emit D rather than D_2_, which is energetically unlikely.

### Simulation results

**Fig. 7:**
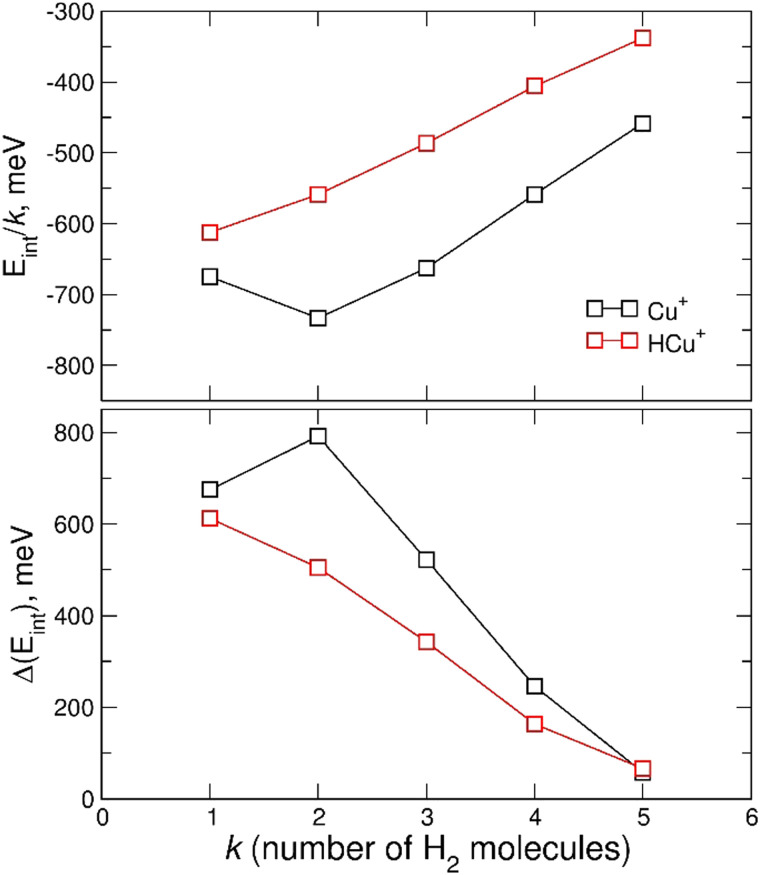
Upper panel: average interaction energy *E*_int_/*k* (in meV) obtained at the CCSD(T)/CBS level for the adsorption of *k* H_2_ molecules around the Cu^+^ and HCu^+^ ions. Lower panel: as above but for the evaporation energy Δ*E*_int_ (in meV).

In Fig. 7 the calculated average interaction energies *E*_int_/*k* are reported as well as the evaporation energies Δ*E*_int_ (defined as *E*^*k*−1^_int_ − *E*^*k*^_int_) related to the optimized global minima structures of the (H_2_)_*k*_Cu^+^ and (H_2_)_*k*_HCu^+^ (*k* = 1–5) clusters given in Fig. 2. It can be noticed that for both species the calculated average interaction energies are higher (more negative) than −300 meV and that the adsorption on the Cu^+^ cation is globally more favorable. Moreover, it is important to stress that the related evaporation energies are quite stronger than those previously predicted for the (H_*2*_)_*k*_Cs^+^ ^45^ and (H_*2*_)_*k*_Na^+^ ^47^ clusters for the same number (*k* < 5) of adsorbed hydrogen molecules. Since for those clusters with alkali monocations the related interaction energy could be described exclusively in terms of noncovalent contributions, it is clear that in the case of the H_2_ adsorption on Cu^+^ and HCu^+^ chemical contributions should play a role. Indeed, a similar behavior was previously found^36^ for the (H_2_)_*k*_Cu^+^ (*k* = 1–4) clusters with large experimental bond dissociation energies (0.67, 0.72, 0.38 and 0.22 eV for *k* = 1–4, respectively) which are in good agreement with present evaporation energies (0.68, 0.79, 0.52 and 0.25 eV for *k* = 1–4, respectively), which predict for *k* = 2 a particularly stable configuration as already evidenced from the above discussion of the experimental data in Fig. 5. Such a behavior is a consequence of the electronic configuration of the involved Cu^+^ and HCu^+^ ions for which the occupied 3d and unoccupied 4s and 4p shells are available to participate in chemical bonding contributions involving the adsorbed H_2_ molecules which can donate the electron charge from the occupied σ orbital.

While 3d and 4s orbital are close, the 4p orbitals are higher in energy with respect to 4s (about 5.5 eV). Since the involved interactions with H_2_ exhibit a strength intermediate between that of canonical chemical and non-covalent cases, it has been suggested that, although the geometries for clusters with *k* = 2, 3, 4 are close to those determined by sp, sp^2^, sp^3^ hybridization of the central ion, it is more effectively controlled by a 3d4s hybridization. In particular, the associated electronic rearrangement stimulates an electron transfer from the occupied σ molecular orbital of hydrogen molecule towards the empty atomic orbitals of central atomic ion which is accompanied by a 3dπ-σ* back-donation.^36^ Both these stabilization effects decrease when *k* becomes larger than 2 and observed findings confirm that canonical hybridizations involving 4s and 4p orbitals, crucial to justify the behavior of complexes of Cu cation with molecules forming strong chemical bonds, here plays only a secondary role.

Further information can be also inferred from the optimized configurations shown in Fig. 2, where the geometries originated by the metal atom and centers of mass of the H_2_ molecules seem to mimic those resulting from different hybridizations of the outer orbital shell of the metal. In fact, (H_*2*_)_*k*_Cu^+^ clusters with *k* = 1–2 show a *C*_2v_ and *D*_2d_ symmetry, respectively, similar to that of a sp-like hybridization; for *k* = 3 the Cu atom and the H_2_ centers of mass lie on the same plane with a *D*_3h_ symmetry; for *k* = 4 a quasi-tetrahedral structure seems to emerge.

On the contrary, for *k* = 5, when an additional H_2_ molecule is added, the inner (H_2_)_4_Cu^+^ structure is practically unchanged while the extra molecule locates itself farther from the metal atom and the corresponding evaporation energy (see lower panel of Fig. 7) strongly reduces down to about 60 meV, that is in the same energy range previously observed for the adsorption on alkali ions, where typical non covalent interactions dominate.^46,47^ Similar considerations also apply to the (H_2_)_*k*_HCu^+^ clusters whose structures show symmetries which could arise from different hybridization of the Cu^+^ orbitals: in this case the pattern as a function of *k* is different (due the presence of a Cu–H chemical bond) and for *k* = 4 a geometry similar to that for a dsp^3^-like hybridization of the metal can be also appreciated (see Fig. 2). An analysis of the related H_2_ internuclear distances and partial atomic charges (obtained by means of the CM5 approach^59^) also evidences differences between the hydrogen molecules pertaining to the first and second shell. In fact, we have checked that for the *k* = 5 cases the outer H_2_ molecule is characterized by an internuclear distance that is shorter of about 0.02 Å; this is compatible with the associated positive partial atomic charges, whose value is around 0.016 a.u. and about 4 times smaller than those associated to the atoms of the innermost H_2_ units, for which a more efficient charge exchange with the Cu ionic core is found.

Therefore, the results in Fig. 7 and Fig. S4 (ESI†), together with the above analysis, confirm that a first solvation shell composed of four H_2_ molecules can be indeed clearly identified. Moreover, this first shell appears to be affected by chemical contributions to the bond in both (H_2_)_*k*_Cu^+^ and (H_2_)_*k*_HCu^+^ (*k* = 1–4) clusters, as shown by the corresponding evaporation energies which largely exceed standard thermal values. Interestingly, even if evaporation energies are in general larger for (H_2_)_*k*_Cu^+^, especially for *k* = 2 and 3, in the case of *k* = 5 we obtain almost coincident values in the range of 60 meV which are in good agreement again with the experimental estimation of ref. 36.

**Fig. 8:**
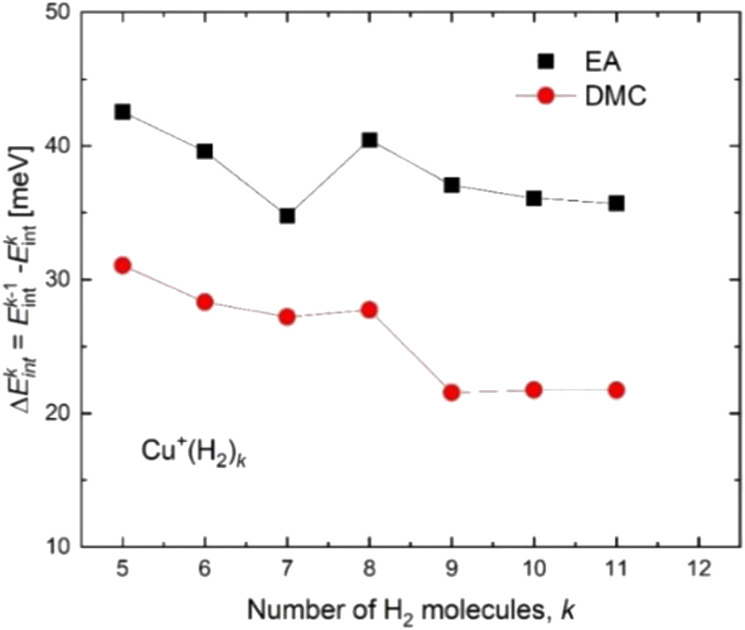
Evaporation energies for (H_2_)_*k*_Cu^+^ obtained by means of the classical EA (black squares) and DMC (red circles) approaches.

Based on the especially stable configuration seen in the *ab initio* calculations for (H_2_)_4_Cu^+^ and following the discussion in the previous sections, we have employed the developed analytical PES to simulate the interaction (basically of non-covalent character) between this structure and the remaining H_2_ units considered in a pseudoatom approximation. This interaction is provided by an ILJ expression shown in eqn (2)–(6) to obtain the evaporation energies for (H_2_)_*k*_Cu^+^ with *k* > 4 by means of a classical optimization EA and a quantum DMC approaches (see ESI,† for details). Results of our theoretical investigation are shown in Fig. 8. The feature found in the evaporation energies for *k =* 8, obtained both by means of the EA and the DMC calculations, suggests that the observed “magic” number in the experimental abundances (see Table 2 and Fig. 5) corresponds indeed to a stable configuration of the external molecular hydrogen which surrounds the (H_2_)_4_Cu^+^ core. This also indicates that a second solvation shell begins at *k* = 5 (as predicted in ref. 36) and probably closes at *k* = 8, which is an exclusive finding of present work.

**Fig. 9:**
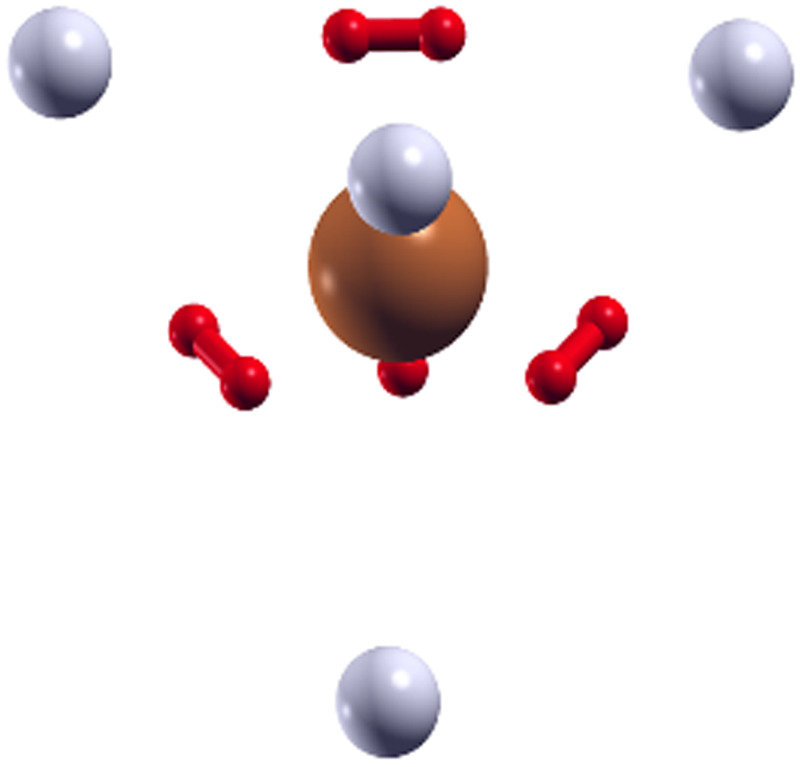
Configuration obtained for the (H_2_)_8_Cu^+^ cluster by means of the EA calculation using the ILJ potential of eqn (1)–(6) where a (H_2_)_4_Cu^+^ rigid core is assumed and the other H_2_ units (here shown as grey spheres) are considered under the pseudoatom approximation.

As shown in Fig. 9, where we present the geometry found for (H_2_)_8_Cu^+^ with the EA calculation employing the analytical PES, the extra four H_2_ units (represented as grey spheres in Fig. 9) are located at approximately 3.4 Å from the Cu^+^ along the axis which connects the center of mass of each inner H_2_ molecule (in red in Fig. 9) with the metal.

### H_*m*_Cu_2_^+^ clusters

**Fig. 10:**
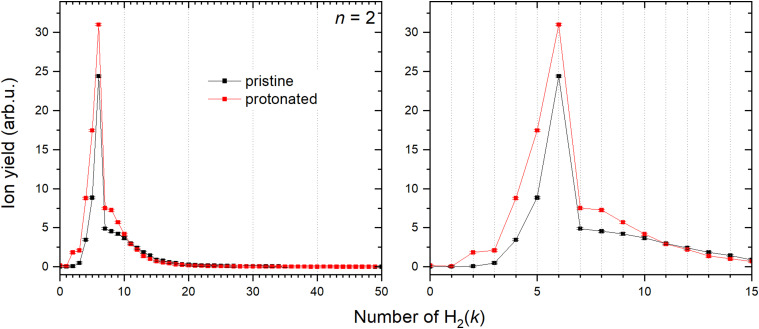
Left: Ion abundances of Cu_2_^+^ (black) and HCu_2_^+^ (red) solvated in the H_2_ with corresponding error bars extracted from the measure mass spectrum. Right: Zoom in on the complexes with first 15 H_2_ molecules attached.

Contrary to Cu^+^, solvation of Cu_2_^+^ shows a very different trend depicted in Fig. 10. Both pristine and protonated clusters show the same “magic” number, for *k* = 6, and additionally HCu_2_^+^ seems to form a stable complex for *k* = 2.

In order to provide an explanation to the presence of the protonated species, an equivalent theoretical analysis as for Cu^+^ (see previous section) has been carried out. A similar conclusion is evidenced with the formation of a very stable HCu_2_^+^ cation, whose optimized structure when interacting with one H_2_ molecule is shown in Fig. 3. Indeed, for the HCu_2_^+^ cation the H atom places itself in between the Cu atoms in a triangular arrangement with a Cu–H bond length of about 1.58 Å and a dissociation electronic energy to form the Cu and HCu^+^ (or Cu_2_^+^ and H) fragments of about 6.0 eV (3.3 eV) is found. Indeed, the ground state of Cu_2_^+^ is thought to exhibit the 3d^20^4σ^1^ configuration, to which corresponds a ^2^Σ_g_ symmetry, and the semi occupied σ orbital can probably receive the electron borne by the H atom to form a covalent bond equally shared by both metal atoms.

**Fig. 11:**
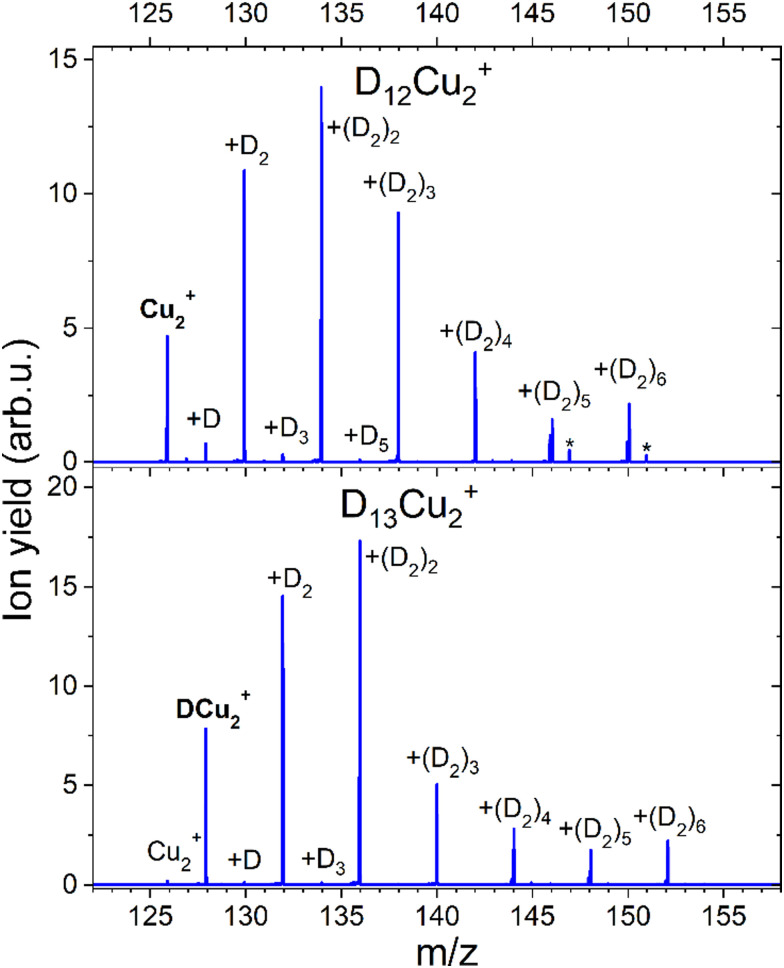
Mass spectra obtained with CID of D_12_Cu_2_^+^ and D_13_Cu_2_^+^ at 10 eV and an Ar pressure of 1 × 10^−5^ mbar. The core ion is indicated with bold text. All further labels indicate how many D atoms are attached to the core ion. Complexes containing D_2_O are marked with an asterisk (*).

CID measurements in Fig. 11 for Cu_2_^+^ were also performed to get the idea about the dissociation pattern of the most pronounced D_*m*_Cu_2_^+^ (*m* = 12, 13) “magic” numbered ions, which correspond to the *k* = 6 case for both pristine and protonated Cu_2_^+^ species. The dissociation pattern in both cases is similar to the one observed for complexes with Cu^+^, that is corresponding to the prevalent detachment of D_2_ units. Interestingly, the complex with *m* = 12, shows some additional peaks (marked with an asterisk) which can be attributed to the D_2_O contamination. Also, for such a complex the loss of a single D atom, which could be a result of water splitting, can be appreciated. Complexes which are formed around the core containing several Cu atoms might not only loose D_2_ but also the metal core might be fragmented if enough energy will be introduced to the system. The CID measurement of D_5_Cu_2_^+^ shown in the Fig. S7 (ESI†) illustrates the changes in the fragmentation pattern as function of energy from 10 to 60 eV. In general, the complex always tends to lose D_2_ molecule, however at energy of 40 eV DCu^+^ and Cu^+^ fragments start to emerge from the noise level together with Cu_2_, whose signal is even lower. When 60 eV is applied it becomes clear that it is more likely to dissociate Cu from the complex rather than the last D atom attached to Cu_2_^+^.

**Fig. 12:**
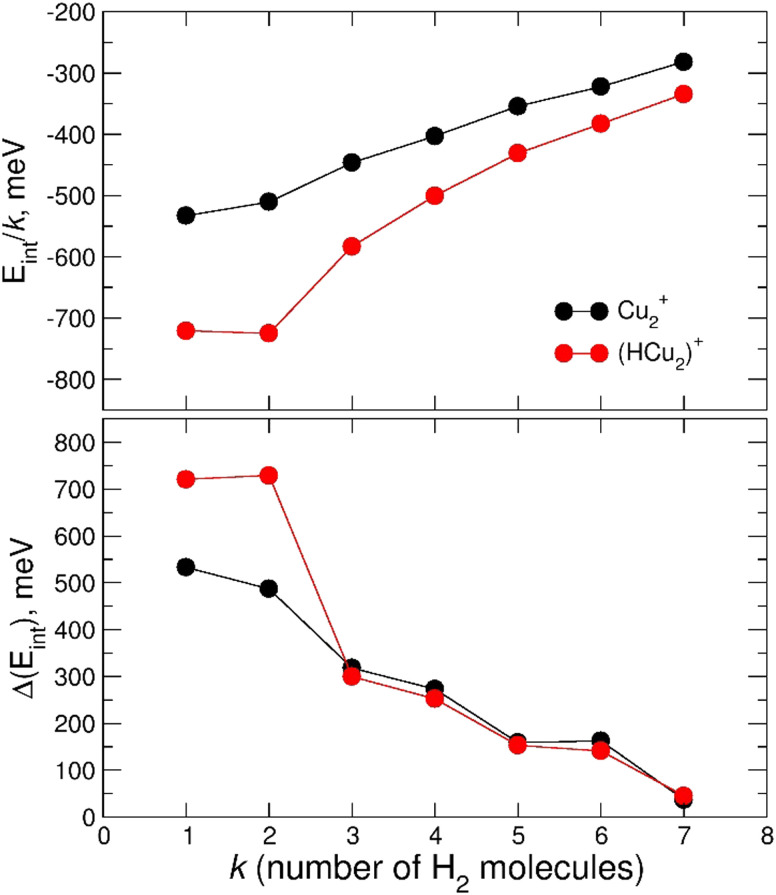
Upper panel: average interaction energy *E*_int_/*k* (in meV) obtained at the CCSD(T)/CBS level for the adsorption of n H_2_ molecules around the Cu_2_^+^ and HCu_2_^+^ ions. Lower panel: as above but for the evaporation energy Δ*E*_int_ (in meV).

The calculated average interaction energies of the (H_2_)_*k*_Cu_2_^+^ and (H_2_)_*k*_HCu_2_^+^ (*k* = 1–7) clusters are reported in Fig. 12. In this case the average interaction energies (see upper panel) are higher (more negative) than −250 meV with adsorption on the protonated cation being more favorable. The latter is probably due to the closed shell character of the HCu_2_^+^ which in general tends to increase the interaction with closed shell species such as H_2_; the same occurs also for the Cu^+^ in Fig. 7 for which a similar trend is observed.

Moreover, in this case the evaporation energies (see lower panel) show a step-like behavior which reflects the tendency to an even distribution of the adsorbed H_2_ molecules on both Cu atoms up to *k* = 6 as shown in Fig. 3. The evaporation energy results seem also to suggest that the (H_2_)_2_HCu_2_^+^ clusters is particularly stable, which is in agreement with the experimental “magic” number reported in Table 2. A comparison between experimental bond dissociation energies (0.54, 0.44, 0.21, 0.16, 0.09 and 0.07 eV for *k* = 1–6, respectively)^37^ for the (H_2_)_*k*_Cu_2_^+^ clusters and present evaporation energies (0.53, 0.49, 0.32, 0.27, 0.16 and 0.16 eV for *k* = 1–6, respectively) shows in general a good agreement. The large binding energies observed and predicted at least for *k* = 1–4 clusters can be interpreted again by invoking chemical contributions mostly involving the donation of the σ orbital electron pair from the adsorbed H_2_. Indeed, the clusters’ geometries reported in Fig. 3 seem to confirm such a behavior and they appear to be the results of a combination of a same or different orbital hybridization of each Cu atom; as an example, for Cu_2_^+^ and *k* = 2, 4 and 6 both metal atoms appear to show a sp, sp^2^, sp^3^-like hybridization, respectively, while for *k* = 3 and 5 the observed structures seem to be originated by a combination of different schemes, *i.e.* sp^2^–sp and sp^3^–sp^2^-like hybridization, respectively. However, when a seventh H_2_ molecule is added it arranges itself farther from the metal atoms, leading to a noticeable decrease of the evaporation energy down to about 40 meV. As in the case of the H_*m*_Cu^+^ clusters (see previous section), an analysis of the H_2_ internuclear distances and atomic charges has been also performed and has confirmed a more efficient charge exchange between the Cu ionic cores and the hydrogen molecules pertaining to the first shell.

Again, these results, together with those in Fig. 3 and Fig. S5 (ESI†), confirm that for the adsorption on Cu_2_^+^ and HCu_2_^+^ cations a first solvation shell composed of six H_2_ molecules can be identified with up to four of them showing a large evaporation energy (>250 meV) clearly exceeding thermal energies.

### H_*m*_Cu_*n*_^+^ (*n* = 3 – 8) clusters

The CID measurements of some complexes with the *n* = 3–5 core at 10 eV, reveal exactly the same pattern as discussed before. Namely, steady loss of D_2_ from the complexes until DCu_*n*_^+^ or Cu_*n*_^+^ is reached for complexes with protonated and pristine cluster cores, respectively. None of the measured structures show any additional peaks as illustrated in Fig. S8 (ESI†) verifying existence of two specific solvation pathways described earlier for Cu^+^ and Cu_2_^+^. Both Cu_*n*_^+^ and HCu_*n*_^+^ cations should therefore coexist and that would explain the high abundance of the (H_2_)_*k*_HCu_*n*_^+^ peak series.

The solvation of the cluster sizes *n* = 3–8 was also studied, found “magic” numbers are listed in the Table 2 and the distributions are shown in the Fig. S9 and S10 (ESI†), which correspond to two different data sets measured under different conditions, proving that the behavior does not depend on the experimental conditions. Cluster sizes with *n* = 3–5 still show several differences in the “magic” numbers between protonated and pristine clusters, while bigger sizes exhibit in both cases a nearly identical behavior.

## Conclusion

Unlike alkali metals (Na^+^ ^47^ and Cs^+^ ^45^) the Cu^+^ cation is found to strongly interact with H_2_ as well as with H species and therefore in this study the role of the solvation of both pristine and protonated Cu^+^ has been investigated. According to previous findings,^36^ the first shell formed by four H_2_ molecules around Cu^+^ is observed. Interestingly, protonated ion HCu^+^ shows the same behavior also requiring four H_2_ units (*k* = 4) to close the first shell and a quasi-tetrahedral structure is formed around both ions. In particular, Cu^+^ shows a closed shell character, due to filled 3d and empty 4s and 4p shells, and in the formation of the bond with H_2_ adopts hybrid atomic orbital that favor the formation of aggregates with H_2_ molecules stabilized by chemical contributions. The latter are due to a partial (perturbative) electron transfer from the occupied σ molecular orbital to the hybrid central ion empty orbital, accompanied by the back donation from the occupied 3dπ to the antibonding σ* of H_2_. Since the first solvation shell, affected by chemical contributions that selectively depend on *k*, is more effectively bound, it remains unaffected by the formation of the second solvation shell, that starts from *k* = 5 and is exclusively controlled by non-covalent interactions. The second solvation shell is found to close at *k* = 8. A similar behavior is found for protonated copper HCu^+^ with a first solvation shell that closes again at *k* = 4 and the second one that closes at *k* = 9.

The binding energy of H_2_ to dimeric copper ions Cu_2_^+^ and its protonated version HCu_2_^+^ is slightly lower than that for the metal monomers, as previously found for sodium clusters.^47^ However, adsorption of H_2_ on the protonated HCu_2_^+^ species is more favorable due to its closed shell character, similarly as was observed for the Cu^+^ monomer. Here again, the first shell is formed due to the donation of electrons from the occupied σ orbital of H_2_ to the empty orbitals of the ions. In this case for both pristine and protonated dimers six H_2_ are required to fill the first shell.

It is found that the evaporation energies associated to the first solvation shell of both Cu monomer and dimer cations clearly exceed thermal values but are also always below 0.8 eV and, therefore, in the right range of interest for hydrogen storage applications, with promising maximum gravimetric capacities varying from about 11 and 8.5 wt%, for (H_2_)_4_Cu^+^ and (H_2_)_6_Cu_2_^+^ clusters, respectively. Moreover, the possible presence of a counterion is expected to decrease the stability of the outer hydrogen shells, due to the reduction of the asymptotic induction attraction controlling the large cationic copper clusters formation, but it should not substantially affect the features of the inner shell.

Additionally, exploiting the high sensitivity of the experimental apparatus we have shown that over 50 H_2_ molecules can be attached to Cu cationic clusters around two different cores Cu_*n*_^+^ and HCu_*n*_^+^, for *n* = 1–8. Experimentally it was shown that H_2_ is strongly bound to the copper core in all cases and these findings were also validated by a theoretical analysis which suggested that for *n* = 1 and 2 the bond between Cu and H_2_ is nearly covalent. When looking at the dissociation patterns formed by the investigated clusters, a similar trend is found for all studied systems. Namely, the detachment of H_2_ units from the complex occurs until the Cu_*n*_^+^ or HCu_*n*_^+^ core is completely stripped, suggesting that the Cu–H bond is much stronger than the bond between HCu^+^ and any of the hydrogen molecules.

To conclude, we believe that the present findings can be relevant not only in the applied field of hydrogen storage and release and catalysis, but also from a fundamental point of view since these clusters offer a unique opportunity to assess the gradual transition between a prevalent covalent bonding, which governs the energy and structure of the first solvation shell found for both Cu monomer and dimer cations, to that of an exclusive non-covalent behavior which determines the intermolecular interaction within the following shells.

## Author contributions

O. V. Lushchikova: conceptualization, data curation, funding acquisition, investigation, project administration, supervision, validation, visualization, writing – original draft preparation. J. Reichegger: investigation, validation. S. Kollotzek: investigation, validation. F. Zappa: investigation, methodology, validation. M. Mahmoodi-Darian: investigation. M. Bartolomei: conceptualization, formal analysis, visualization, writing – original draft preparation. J. Campos-Martínez: investigation, funding acquisition, writing – review & editing. T. González-Lezana: investigation, funding acquisition writing – review & editing. F. Pirani: conceptualization, investigation, methodology, writing – review & editing P. Scheier: conceptualization, funding acquisition, resources, methodology, conceptualization, software, writing – review & editing.

## Conflicts of interest

There are no conflicts to declare.

## Supplementary Material

CP-025-D3CP03452F-s001
