# Supplementary material for: Solvation of cationic copper clusters in molecular hydrogen
Source: Phys Chem Chem Phys. 2023 Sep 13;25(37):25251–63. doi: 10.1039/d3cp03452f (PMC10528801; doi:10.1039/d3cp03452f)
Supplement: CP-025-D3CP03452F-s001 [file CP-025-D3CP03452F-s001.pdf]

Electronic Supplementary Material (ESI) for Physical Chemistry Chemical Physics.
This journal is © the Owner Societies 2023

Solvation of cationic copper clusters in molecular hydrogen

O.V. Lushchikova[1*], J. Reichegger[1], S. Kollotzek[1], F. Zappa[1], M. Mahmoodi-Darian[2], M. Bartolomei[3*], J. Campos-Martínez[3], T. González-Lezana[3], F. Pirani[4], P. Scheier[1]

[1] Institut für Ionenphysik und Angewandte Physik, Universität Innsbruck, Technikerstraße 25, Innsbruck 6020, Austria
[2]Department of Physics, Karaj Branch, Islamic Azad University, Karaj, Iran
[3] Instituto de Física Fundamental, IFF-CSIC, Serrano 123, Madrid 28006, Spain
[4]Dipartimento di Chimica, Biologia e Biotecnologie, Universita´ di Perugia, 06123 Perugia, Italy

Supplementary Information

This Supplementary Information contains:

1. $H_mCu_n^+$ distributions and experimental details
2. $(H_2)_4Cu^+$ minimum geometry
3. Collision induced dissociation measurements
4. Ion abundances
5. Simulation methods

1. $H_mCu_n^+$ distributions and experimental details

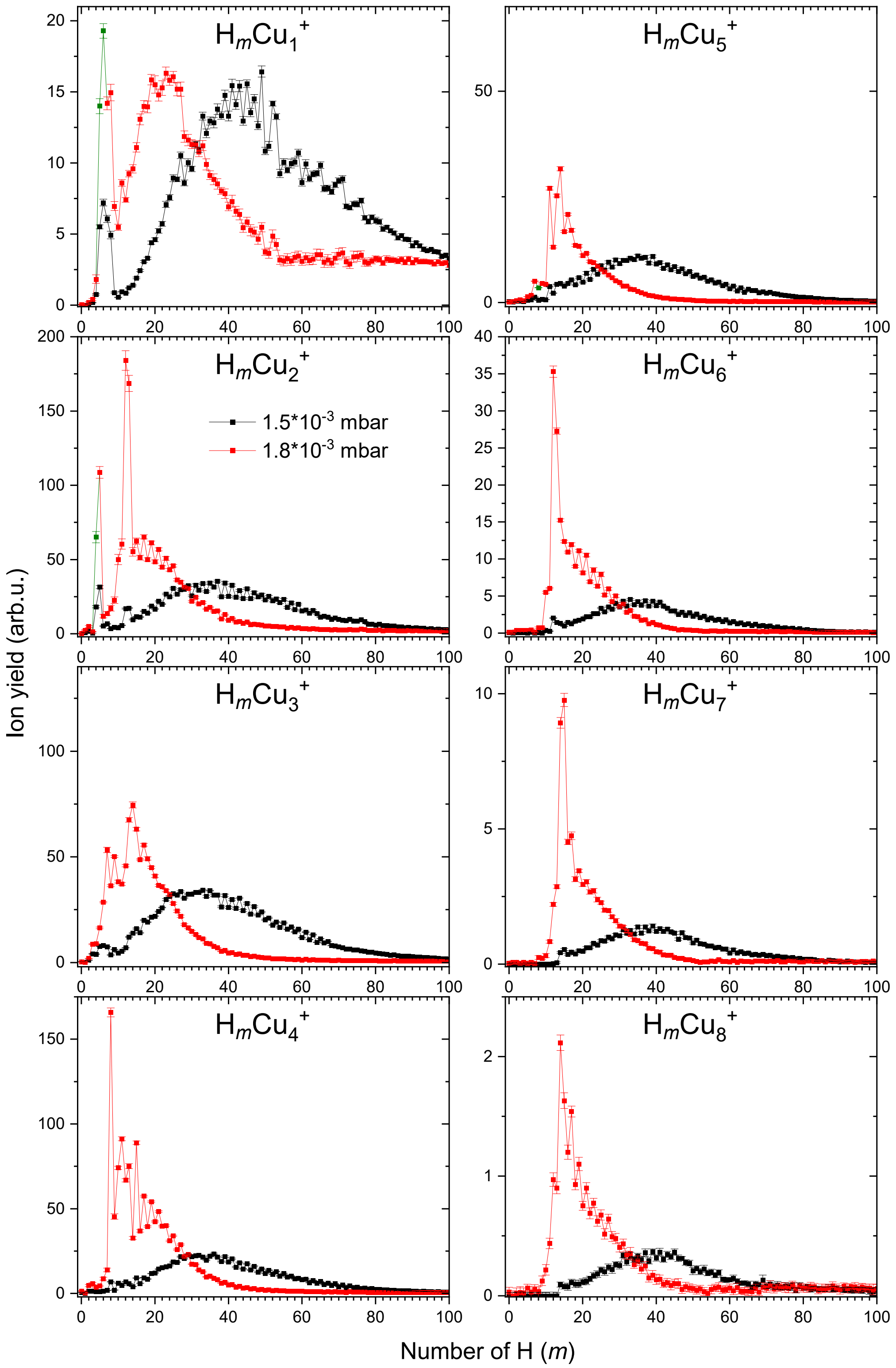

Figure S1: $H_mCu_n^+$ distributions as a function of the number of H atoms (*m*) adsorbed to each individual cluster size (*n*) at H_2 pressure in the hexapole chamber of 1.5×10^{-3} (black) and 1.8×10^{-3} (red) mbar. Measurements are done at conditions specified as "H_2 big clusters" in table S1.

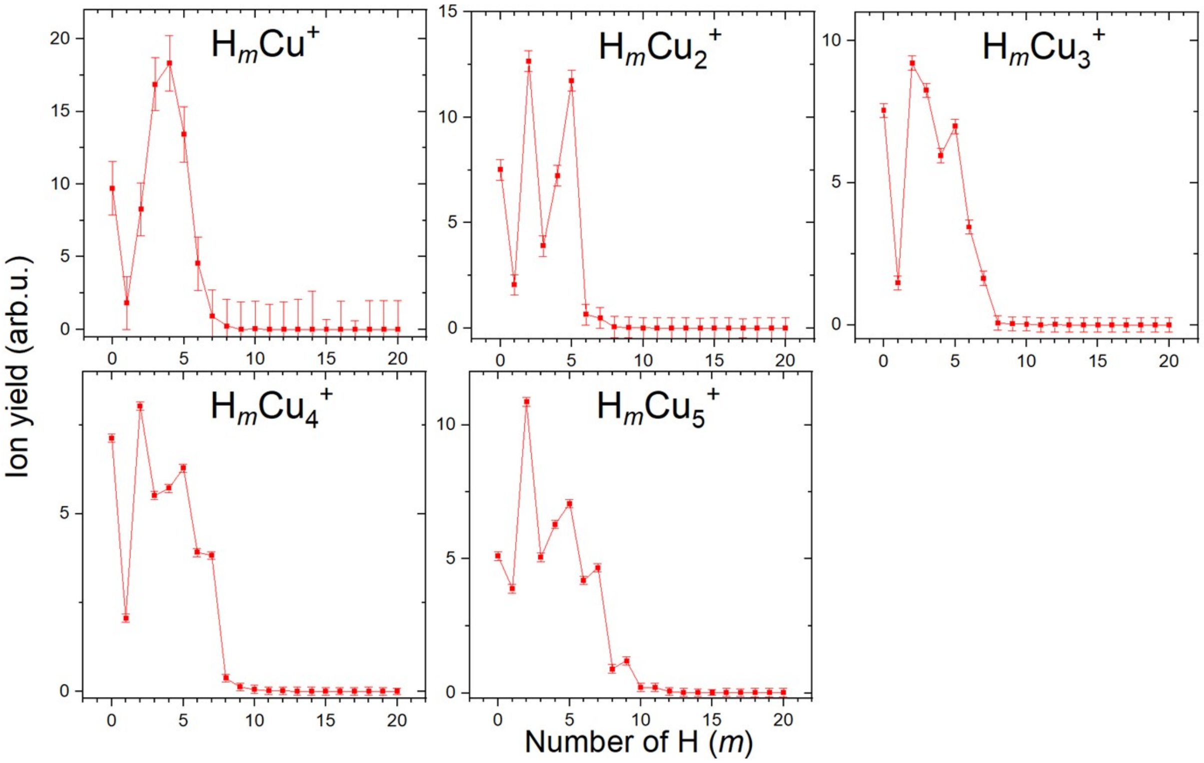

Figure S2: $H_mCu_n^+$ distributions as a function of the number of H atoms (m) adsorbed to copper cluster cations with a size (n) between 1 and 5. The complexes are formed by introducing H_2 to the quadrupole chamber prior Cu cluster formation) to the same pressure as was used during measurements in figure S1. To shrink He droplets H_2 was replaced by the room temperature He.

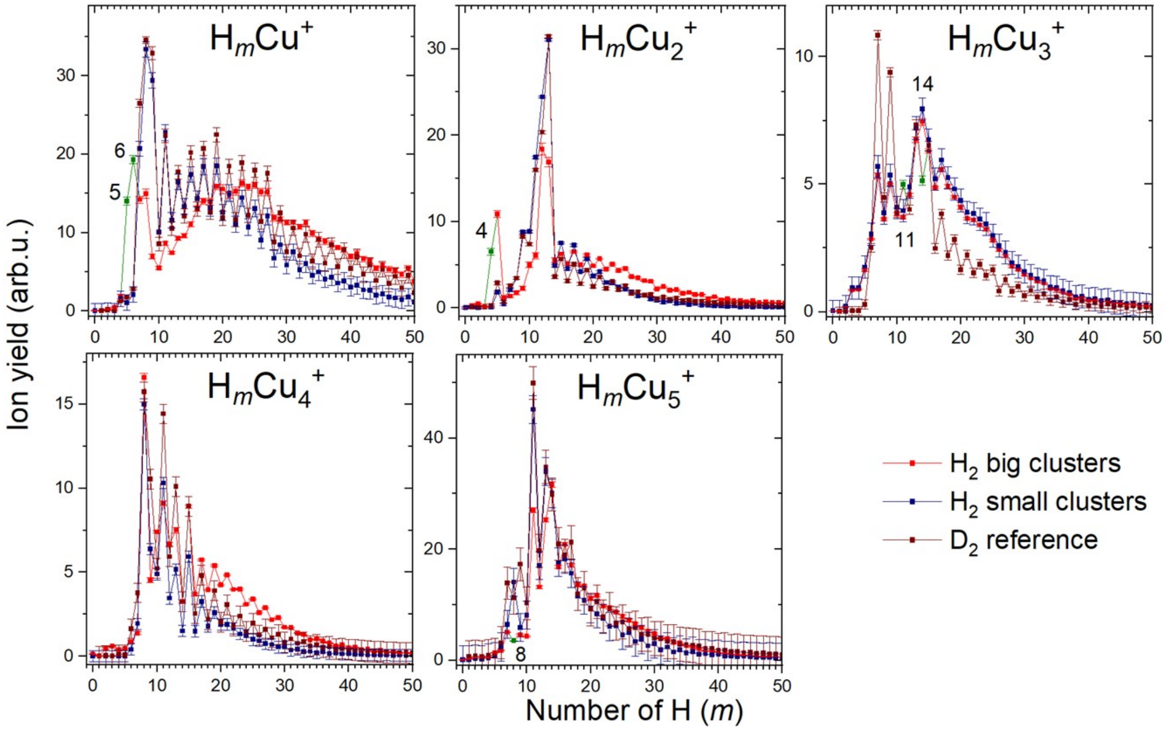

Figure S3: The measurement from figure S1 obtained at a H_2 pressure of 1.8×10^{-3} (red) mbar is verified with two different reference measurements measured with different conditions using H_2 (blue) and D_2 (brown). The conditions of all three measurements are listed in table S1. Only complexes with n = 1-5 are compared, since bigger clusters were not produced with conditions "H_2 small clusters". The data points which do not follow the general trend are marked green.

Table S1: Experimental conditions used to obtain data in figure S3.

	H_2 big clusters	H_2 small clusters	D_2 reference
**He Pressure [Bar]**	31	19	25
**Nozzle Temperature [K]**	9.3	7	7
**Quad Pressure [mbar]**	6.20×10^{-7}	1.10×10^{-6}	8.60×10^{-7}
**Ion Block Potential [V]**	245	375	319
**Deflector Vertical [V]**	9.7	2.4	5.3
**Deflector Horizontal [V]**	9	4.8	9.5
**Electron Energy [eV]**	56	60	73
**Electron Current [Ua]**	370	280	220
**Float Voltage [V]**	-140	-387	-459
**Deflect Voltage [V]**	-275	-280	-315
**Float Z [V]**	45	37	32
**U Z [V]**	15	18	25
**Float Y [V]**	-24	35	79
**U Y [V]**	-25	32.3	25.4
**Front Aperture [V]**	0	-470	-473
**Oven Type**	shapal	shapal	shapal
**Oven Voltage [V]**	7.54	7.84	8.77
**Oven Current [A]**	15.23	15.3	16.55
**Oven Power [W]**	115	120	145
**Pickup Pressure [mbar]**	1.14×10^{-5}	2.7×10^{-5}	2.1×10^{-5}
**Evaporation Gas**	H_2	H_2	D_2
**Pressure [mbar]**	1.83×10^{-3}	1.83×10^{-3}	2.72×10^{-3}

2. $(H_2)_4Cu^+$ minimum geometry

Table S2: $(H_2)_4Cu^+$ minimum geometry and charge distribution

Atom	x(Å)	y	z	q(a.u.)
**Cu**	0	0.01311	0	0.87
**H**	0.12480	-1.87601	0	0
**H**	0.87636	-1.66331	0	0
**H**	0.45815	0.49696	1.71717	0
**H**	0.85686	1.01778	1.28614	0
**H**	0.45815	0.49696	-1.71717	0
**H**	0.85686	1.01778	-1.28614	0
**H**	-1.81559	0.06489	0.39175	0
**H**	-1.81559	0.06489	-0.39175	0

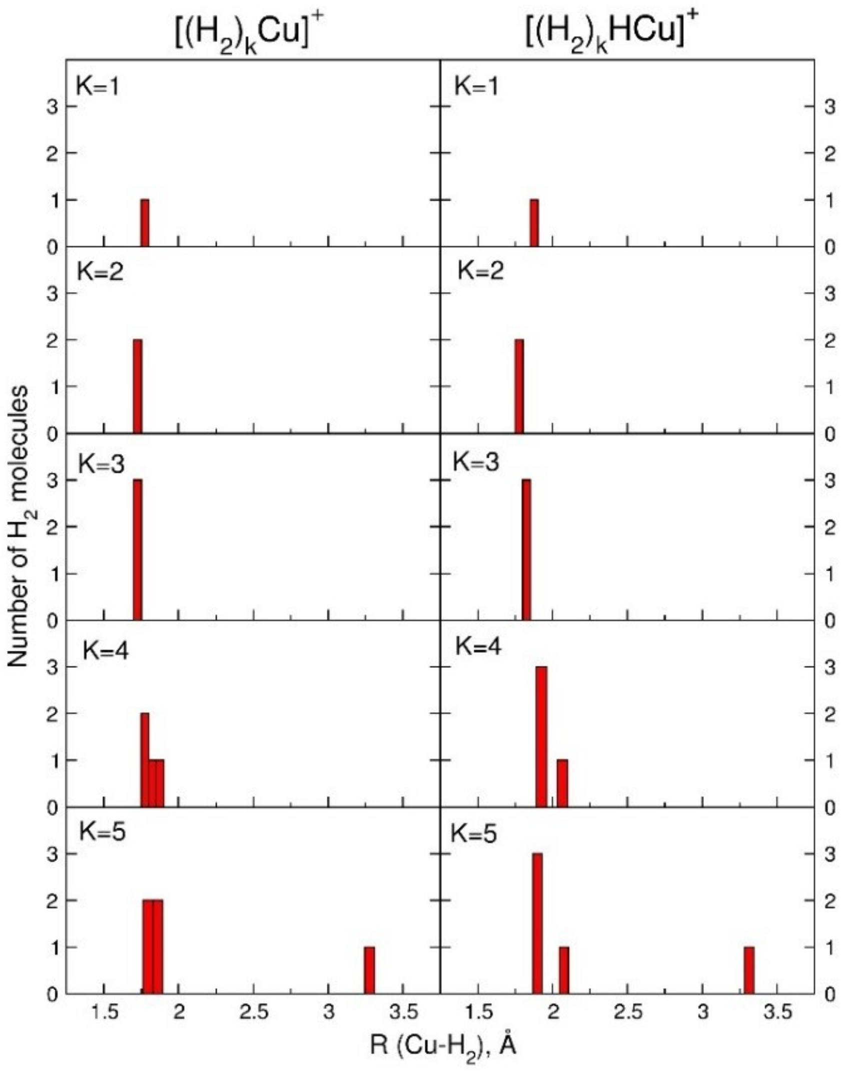

Figure S4: Histograms for the separation distance R of the H_2 centers of mass from the Cu atom for the optimized geometries of the $(H_2)_kCu^+$ and $(H_2)_kHCu^+$ (k = 1-5) clusters depicted in figure 2 of the main text.

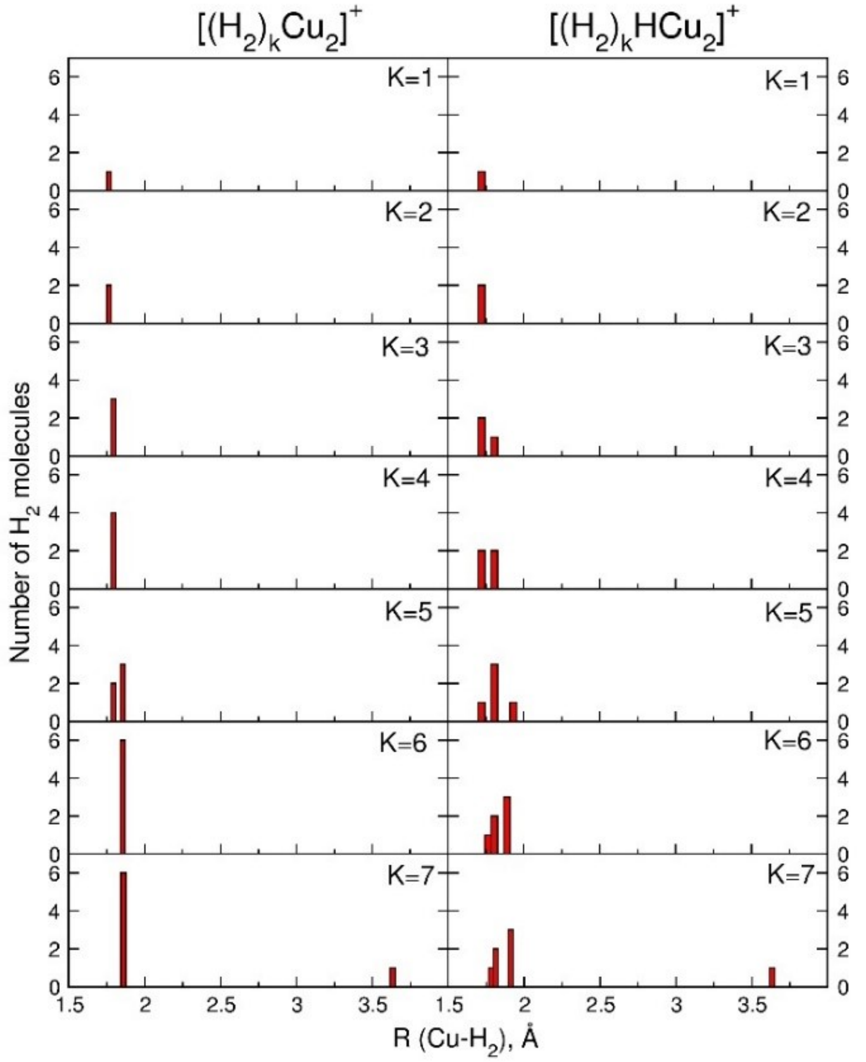

Figure S5: Histograms for the separation distance R of the H_2 centers of mass from the Cu atoms for the optimized geometries of the $(H_2)_kCu_2^+$ and $(H_2)_kHCu_2^+$ (k = 1-7) clusters depicted in figure 3 of the main text.

3. Collision induced dissociation measurements

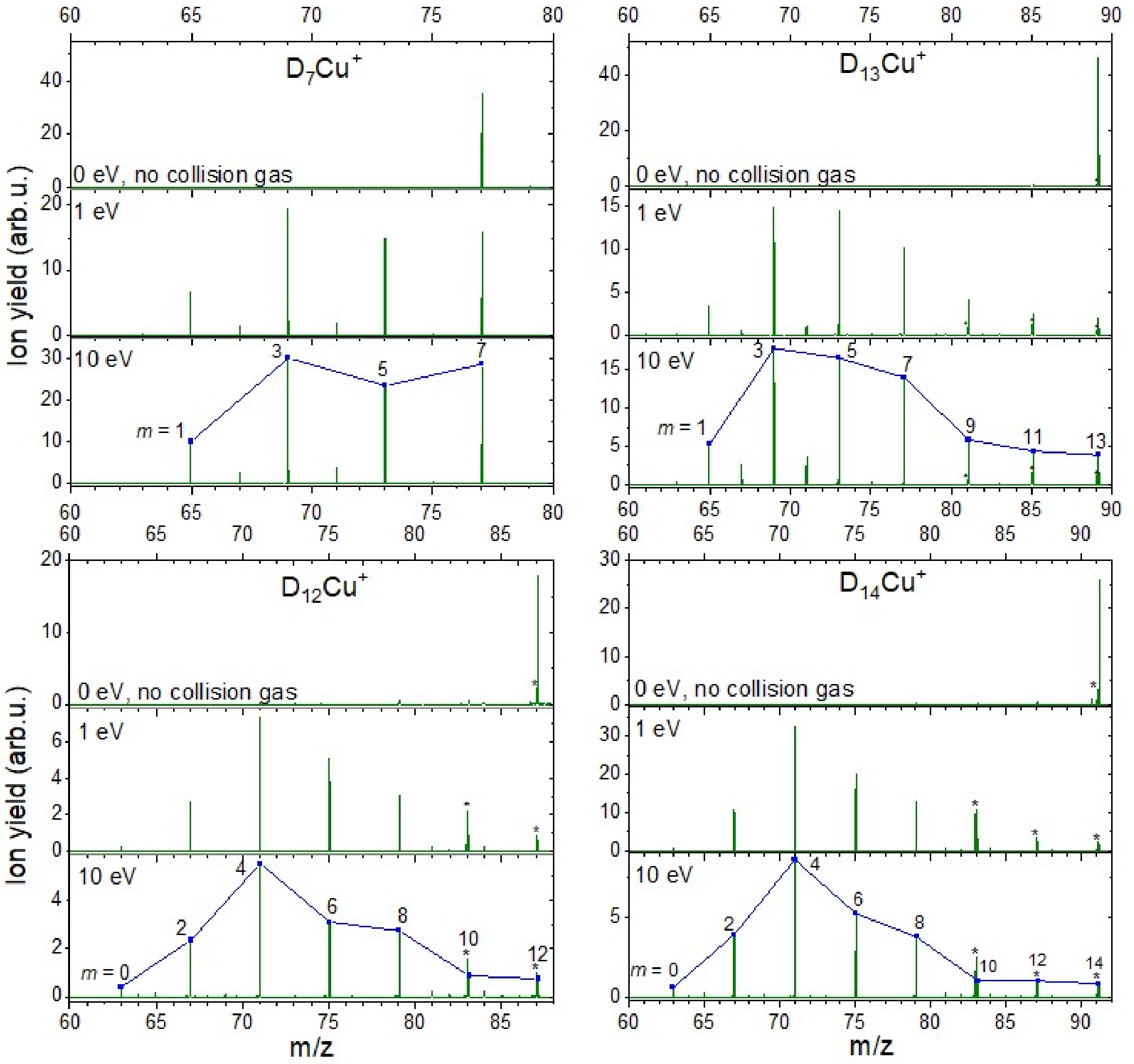

Figure S6: CID measurements of Cu^+ with 7, 12, 13 and 14 D atoms attached. For each complex three measurements were done at 0 eV (without any collision gas), 1 and 10 eV (with Ar as a collision gas). The main fragmentation path is represented in blue and the number of D atoms attached to Cu^+ are also indicated. The peaks originated from the complexes with D_2O impurity are marked with the asterisk.

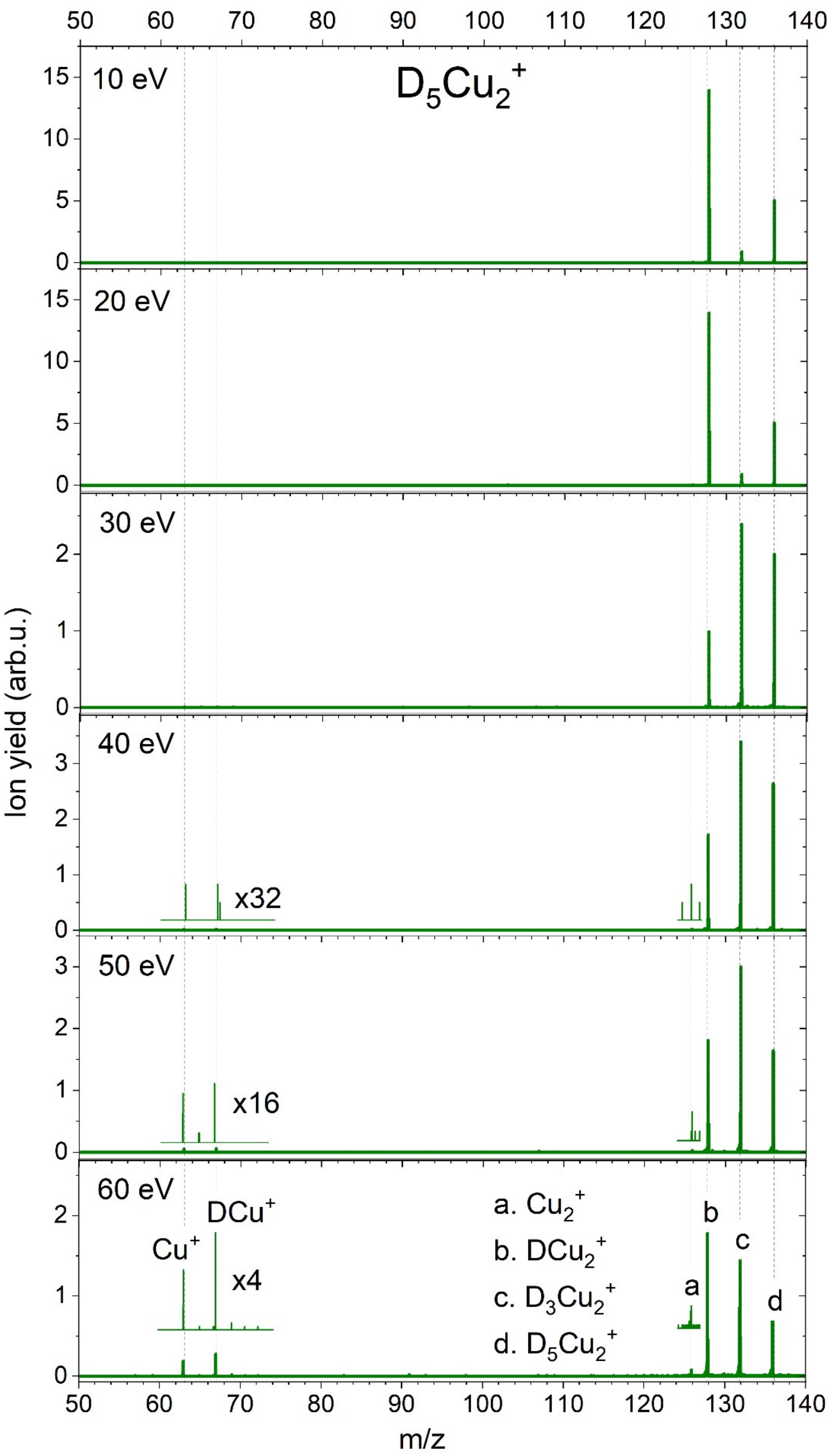

Figure S7: CID measurements of $D_5Cu_2^+$ complex at the energy of 10-60 eV in steps of 10 eV. The zoom-in on the Cu^+, D_2Cu^+ and DCu_2^+ fragments at energies >30 eV is also shown with the corresponding magnifying factor.

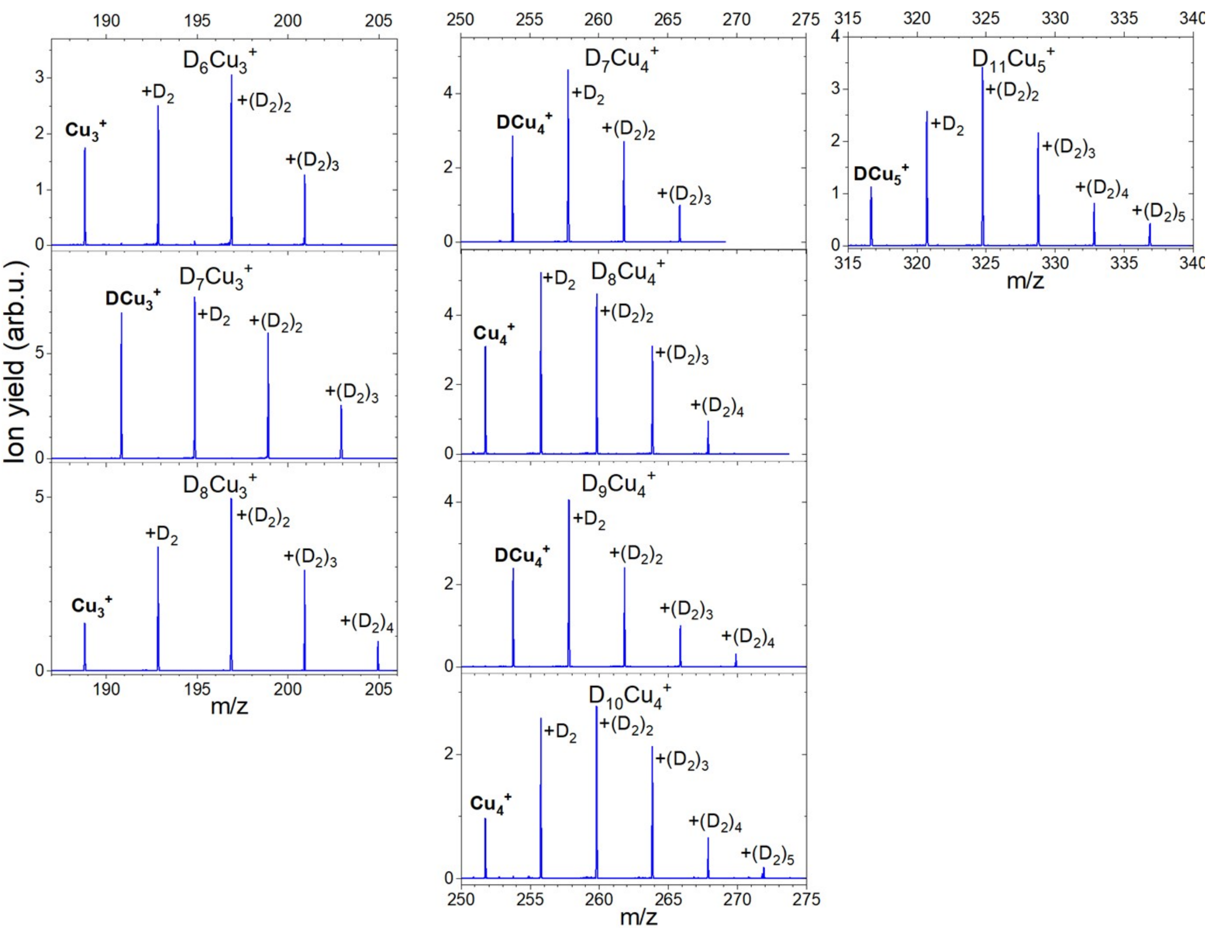

Figure S8: CID of some selected “magic” ions for *n*=3-5 measured at 10 eV.

4. Ion abundances

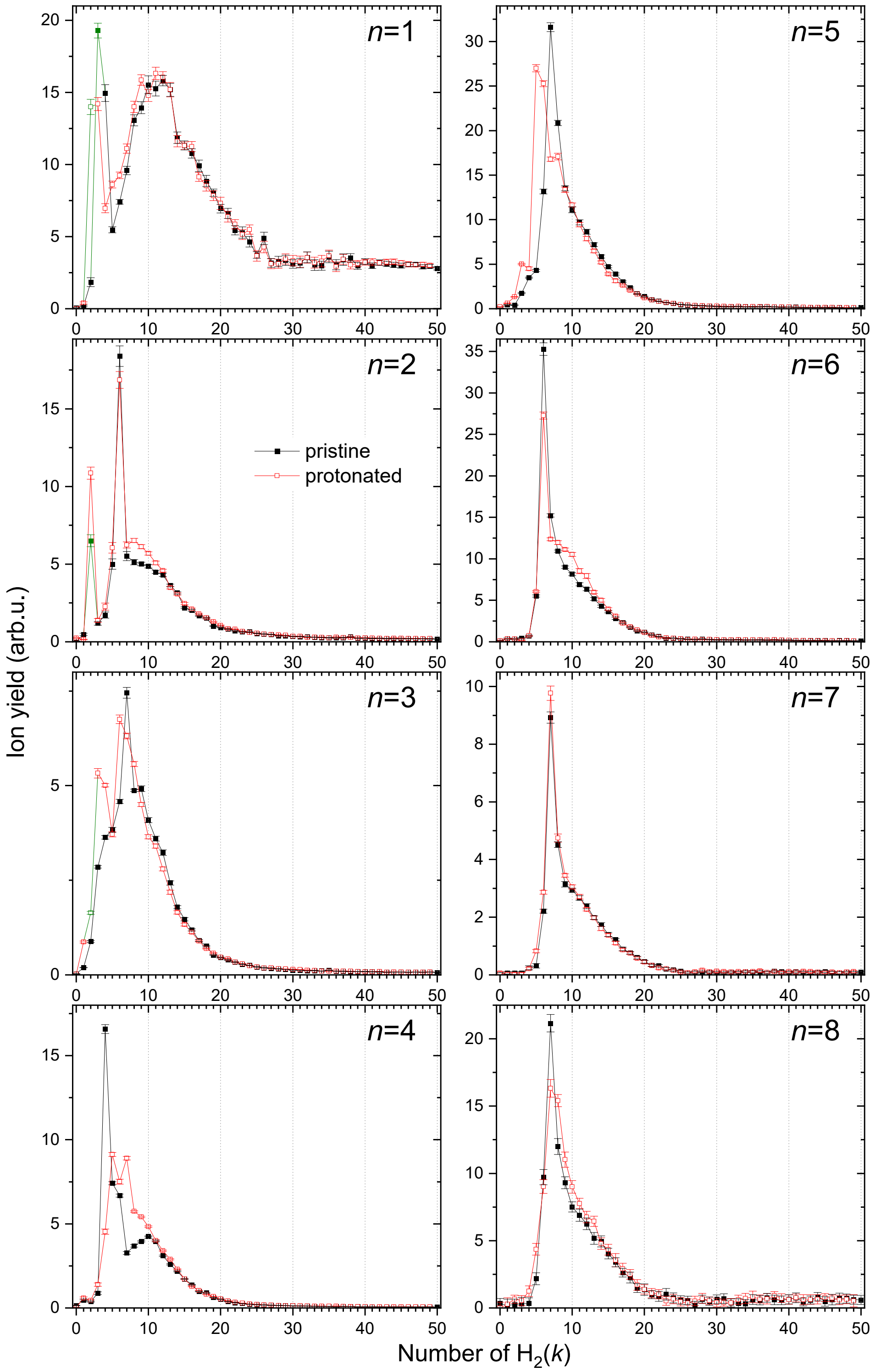

Figure S9: Ion abundances of Cu_n^+ (black) and HCu_n^+ (red) for n=1-8 solvated in the H_2 with corresponding error bars extracted from the mass spectrum measured at the "H_2 big clusters" conditions from table S1. The ion abundances are plotted for each separate cluster size as a function of H_2 (k). The measurement points highlighted in green were not verified by the reference measurements and therefore are not considered.

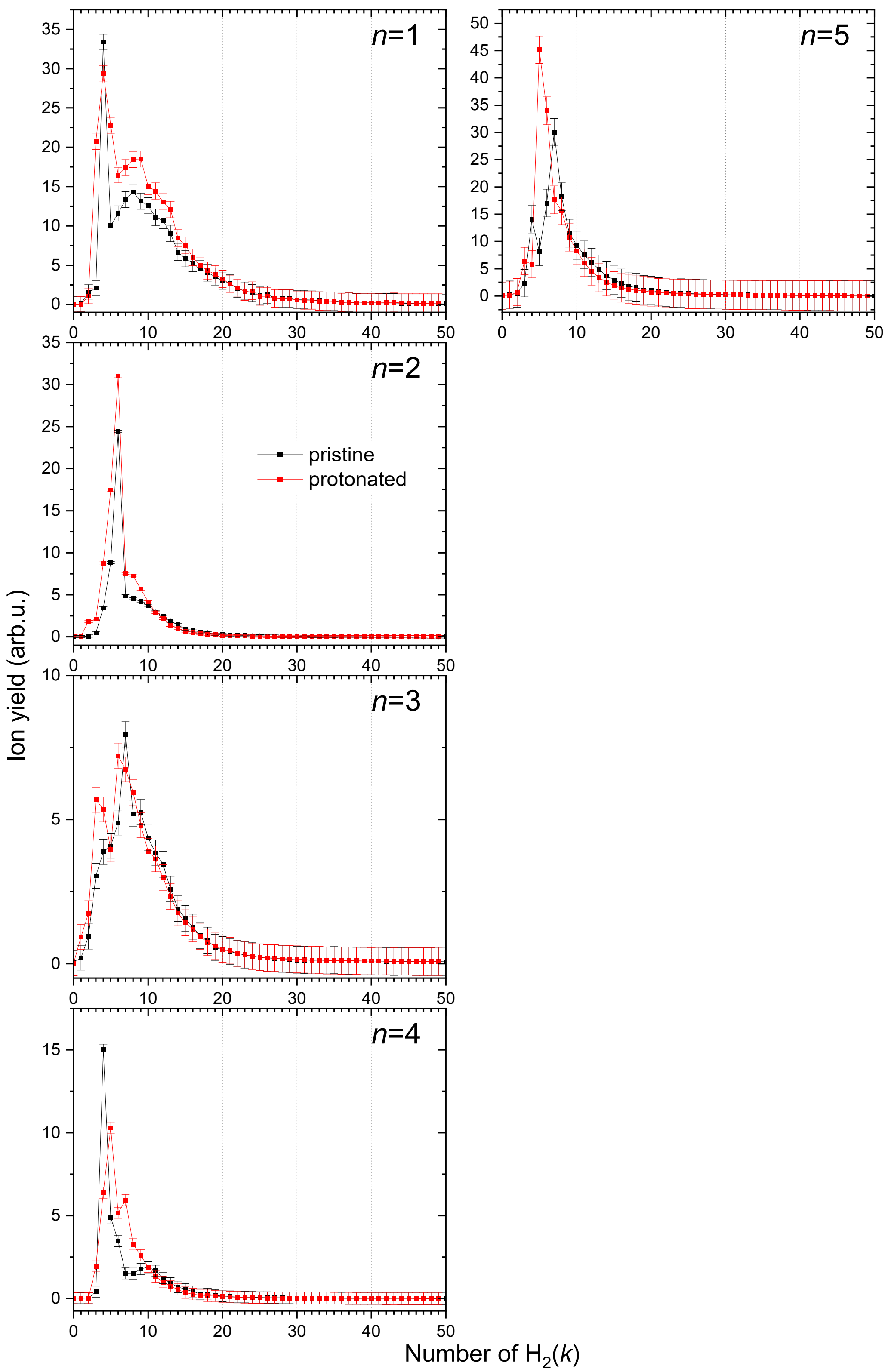

Figure S10: Ion abundances of Cu_n^+ (black) and HCu_n^+ (red) for n=1-5 solvated in the H_2 with corresponding error bars, similar to figure S7, but measured at the “H_2 small clusters” conditions from table S1. The ion abundances are plotted for each separate cluster size as a function of H_2 (k).

5. Simulation methods

In this work we have employed the evolutionary algorithm (EA) and a diffusion Monte Carlo (DMC) method to study both the energy and structure of the $(H_2)_kCu^+$ (k>4) clusters following the description discussed in the main text: The first four H_2 units are assumed as fixed as the global minimum shown in Figures 1 and 3. The other H_2 units are considered as pseudoatoms and their interaction with the inner $(H_2)_4Cu^+$ core is described by means of the potential shown in Eqs. (3) – (5)

5.1 Evolutionary Algorithm

The so called EA [1] provides insights of the minimum energy configurations of the $(H_2)_kCu^+$ clusters. This algorithm has already been used successfully for doped hydrogen [2] and helium [3] clusters. The method starts with *M* (equals to 30 in our calculation) parent populations which are confronted as in a natural selection procedure with offspring populations obtained after inducing some mutations in the original ones. The conformational space of the system is then explored through the optimization of a fitness function to search for the overall minimum energy. Groups of 10 individuals are confronted and the best fit (within an energy threshold of 10^{-4} meV). In the present study we start generating initial populations of *M* clusters consisting of *N* H_2 units treated as pseudoatoms. surrounding the $(H_2)_4Cu^+$ core. Each individual *i* is characterized by the pair of vectors ($\hat{z}_i$, $\tilde{\eta}_i$) representing the 3*N* Cartesian coordinates of the atoms and standard deviations for Gaussian mutations, respectively. We start with $\eta_i = 1$ and with random selections for the position within a specific range (0, Δ). A single offspring (z'_i, η'_i) is created for each parent following the conditions:

$z'_i(j) = z_i(j) + \eta_i(j)$

$\eta'_i(j) = \eta_i(j) \exp[\tau' N(0,1) + \tau N_j(0,1)]$

where j = 1, …, 3*N*; τ and τ' are adjustable parameters which depend on the value of *N*; N(0, 1) is a random number from a Gaussian distribution of mean μ = 0 and standard deviation σ = 1, and $N_j(0, 1)$ stands for a randomly generated number for each component j.

The following step is then to establish pairwise comparisons of the energy of each individual with *q* random choices as opponents over the union of 2M elements formed with parents (z'_i, η'_i) and offsprings (z'_i, η'_i). We select those M individuals from the total population formed both with parents and offsprings with a larger number of points awarded in the competition with some other opponents to search for the lowest energies. These survivors individuals thus become parents for a new iteration in the selection process which will be repeated until the difference between the potential energies of consecutive generations is lower than a certain selected tolerance value.

5.2 Diffusion Monte Carlo

We use the DMC approach [4,5] to obtain the ground state energies, and the corresponding probabilities and distributions. In this method the time-dependent Schrödinger Equation is transformed in a diffusion equation by the change τ=i t. The ground state will correspond with the last non vanishing term in the propagation of the diffusion equation. We have used

the code developed by Sandler and Buch[6,7]. The calculations were made with time step sizes, Δτ, ranging between 12.5 and 100.0 a.u. and a number of steps from 40000 to 5000 up to reach convergence. The number of replicas (or walkers) accounted for in all calculations were between 25000 and 29000. The number of seeds were 8, and the probability density was obtained with 8 generations for each DMC run. With these parameters the statistical errors in energies were in the (0.01, 0.12) range.

[1] M. Iwamatsu, *Comput. Phys. Commun*., 2001, **142**, 214–218

[2] S. Kollotzek, J. Campos-Martínez, M. Bartolomei, F. Pirani, L. Tiefenthaler, M. I. Hernández, T. Lázaro, E. Zunzunegui-Bru, T. González-Lezana, J. Bretón, J. Hernández-Rojas, O. Echt and P. Scheier, *Phys. Chem. Chem. Phys*. 2023, **25**, 462.

[3] E. Zunzunegui-Bru, E. Gruber, T. Lázaro, M. Bartolomei, M. I. Hernández, J. Campos-Martínez, T. González-Lezana, S. Bergmeister, F. Zappa, R. Pérez de Tudela, J. Hernández-Rojas, J. Bretón, *J. Phys. Chem. Lett*. 2023, **14**, 3126.

[4] J. B. Anderson. J. Chem. Phys. 1975, 63, 1499.

[5] M. A. Suhm, R. O. Wattsm, Phys. Rep. 1991, 204 293.

[6] V. Buch, J. Chem. Phys. 1992, 97, 726.

[7] P. Sandler, V. Buch, J. Sadlej, J. Chem. Phys. 1996, 105, 10387.
